# Antimicrobial stewardship implementation before and during the COVID-19 pandemic in the acute care settings: a systematic review

**DOI:** 10.1186/s12889-023-15072-5

**Published:** 2023-02-10

**Authors:** Rasha Abdelsalam Elshenawy, Nkiruka Umaru, Amal Bandar Alharbi, Zoe Aslanpour

**Affiliations:** Department of Clinical, Pharmaceutical and Biological Sciences, University of Hertfordshire School of Life and Medical Sciences, Hatfield, AL10 9AB UK

**Keywords:** Antimicrobial stewardship strategies, Antimicrobial stewardship measures, Antimicrobial resistance, Coronavirus, COVID-19 pandemic, Acute care settings

## Abstract

**Introduction:**

Antibiotics are widely administered for various indications, leading to increased antimicrobial resistance (AMR) in acute care hospitals. Since the onset of the COVID-19 pandemic, Antimicrobial Stewardship (AMS) effective strategies should be used to maintain the rational use of antibiotics and decrease the threat of Antimicrobial Resistance (AMR).

**Aim:**

This systematic literature review aims to investigate the AMS intervention Before-the-pandemic (BP) and During-the-pandemic (DP) from the literature.

**Design and setting:**

Systematic literature review of primary studies on AMS implementation in acute care settings.

**Methods:**

Relevant studies published between 2000 and March 2021 were obtained from Medline (via PubMed), OVID, CINAHL, International Pharmaceutical Abstracts, Psych Info, Scopus, Web of Science, Cochrane Library, OpenGrey, and Google Scholar, using a comprehensive list of search terms. Public Health England (PHE) toolkit was agreed upon as a gold standard for the AMS implementation.

**Results:**

There were 8763 articles retrieved from the databases. Out of these, 13 full-text articles met the inclusion criteria for the review. The AMS implementation was identified in the included studies into AMS strategies (Core strategies & Supplemental strategies), and AMS measures BP and DP.

**Conclusion:**

This Systematic literature review summarises AMS implementation strategies and measures all over the previous 20 years of research. There are many lessons learnt from COVID-19 pandemic. The proper selection of the AMS implementation strategies and measures appeared to be effective in maintaining the appropriate use of antibiotics and decreasing the AMR threat, especially during the COVID-19 pandemic. Further studies are required to provide empirical data to evaluate the AMS implementation and identify which of these strategies and measures were effective BP and DP. In order to be prepared for any emergency/crisis or future pandemics.

**Supplementary Information:**

The online version contains supplementary material available at 10.1186/s12889-023-15072-5.

## Introduction

Sir Alexander Fleming mentioned the concept of Antimicrobial Resistance (AMR) during his Nobel Prize lecture [1]. The rise in multi-drug-resistant infections threatens global health through significant morbidity, mortality, and economic loss. Following the O’Neill review and findings in 2016, the number of deaths from AMR infections was estimated to reach 10 million annually due to the AMR crisis [2]. AMR is a silent pandemic and one of the biggest threats to global health [3]. In 2019, it was estimated that more than 1.2 million people died worldwide from AMR [4].

Public Health England (PHE) has also emphasised the need for AMS implementation to maintain the appropriate use of antibiotics [5]. Antimicrobial Stewardship (AMS) is a coherent set of actions that promotes the effective use of antibiotics. It aims to maintain the optimal selection, dosage, route, and duration of antibiotic treatment [6]. For more definitions of Antimicrobial Stewardship, see Supplementary Table S1. Many AMS strategies are used to maintain the judicious use of antibiotics and educate prescribers. Furthermore, the AMS implementation should be measured in order to evaluate the outcomes of AMS implementation [7, 8].

The outbreak of infection caused by the severe acute respiratory syndrome coronavirus-2 (SARS-CoV-2; COVID-19) from Wuhan, China, in December 2019 escalated rapidly to become a global pandemic [9]. In June 2022, the global estimate for people who tested positive for COVID-19 was approximately 544 million. Additionally, the estimated number of total deaths is 6 million, 10% of the worldwide deaths of 60 million [10]. Recent evidence suggests that, as a consequence of the COVID-19 pandemic, increasing numbers of patients admitted to hospitals have been prescribed empirical antimicrobial therapy, which may not always be appropriate, potentially increasing the number of resistant infections globally [11, 12]. While consideration for AMR and AMS focused on supporting the selection of optimal empirical therapies and appropriate de-escalation or discontinuation of antimicrobials when bacterial co-infection is present or absent is essential [13].

Indeed, results from one of the previously published systematic reviews suggested that co-infection prevalence with resistant bacterial organisms was 24%. Sadly, of the 1959 unique isolates identified within the included studies, 569 (29%) were deemed resistant [11]. Another systematic review and meta-analysis also found an overall high antimicrobial consumption among COVID-19 patients [14]. However, the AMS intervention during the COVID-19 pandemic within a systematic review has not been published to date. A critical knowledge gap exists regarding the AMS implementation strategies DP in acute care settings. This systematic review addressed the research question: “What are the AMS implementation strategies and measures?” The objectives were to (1) review AMS before and during the COVID-19 pandemic; (2) assess the acute care settings and geography; (3) document AMS strategies and measures if available, and (4) estimate the proportion of each strategy and measures reported in the literature.

## Materials and methods

### Registration

Prior to the initial search, the review was registered at PROSPERO (registration number CRD42021242388) [15]. The scope of the review was defined by applying the acronym PICOS (Population, Intervention, Comparison, Outcome, Setting), as shown in Table 1. A systematic search of databases was conducted using the following keywords and their synonyms (for more details, see Supplementary Tables S2 and S3). After this, follow the Preferred Reporting Items for Systematic Reviews (PRISMA) guidelines for reporting. The PRISMA 2020 was drawn up and approved by the research team before the commencement of the systematic review [16]. The plan was employed as a guidance document to systematically review relevant primary studies published between 2000 and 2021. It described the review's scope, intended purpose, and methodological and analytical approach. Ethical approval was not required before the commencement of the review as the use of patients’ identifiable data was not intended.

**Table 1:** Inclusion and exclusion criteria

	**Inclusion criteria**	**Exclusion criteria**
**Participants**	Studies targeting the public/patients’ use of antibiotics Healthcare Professionals (HCPs) who are responsible for prescribing, dispensing, or administering antibiotics (doctors, pharmacists)	Non-HCPs (patient family or community or nursing or long-term care patients)
**Intervention**	Studies describe an intervention to improve antibiotic prescribing or AMS or any other intervention as the use of the parenteral-to-oral switch and the duration of IV and oral antibiotics	Studies that do not describe an AMS intervention
**Comparison**	Comparison with a control group/a group that carried out usual care without an AMS intervention; comparison between two or more AMS interventions	
**Context**	Interventions carried out in adult inpatient settings in acute care hospitals	Interventions carried out in nursing homes, care homes or long-term healthcare facilities; community settings; paediatric setting/hospital; and animals/ veterinary practice
**Outcomes**	Primary outcomes: reviewing the AMS implementation before and during the COVID-19 pandemic	
	Secondary outcomes: other AMS measures, metrics, and quality improvement before and during the COVID-19 pandemic	
**Study design**	Randomized Controlled Trials (RCTs), non-randomized trials, Controlled Before-After (CBA) studies, interrupted time series designs, case–control and cohort studies, cross-sectional studies, and qualitative studies	Literature reviews, systematic reviews, meta-analyses, single case studies, case reports, and conference abstracts

(a) *HCPs* Healthcare Professionals, *AMS* Antimicrobial Stewardship, *COVID-19* Coronavirus

(b) *RCTs* Randomized Controlled Trials, *CBA* Controlled Before-After

### Eligibility screening

The articles retrieved from the databases were exported into CSV and Excel sheets for screening and identification of the eligible articles by RAE. Titles and abstracts were screened for relevance; duplicates were removed, followed by a screening of the complete articles for possible inclusion by one reviewer (RAE). Another reviewer (ZA) independently reviewed the titles, abstracts, and full studies, confirmed the relevance of studies in meeting the inclusion criteria and excluded studies deemed irrelevant. Three reviewers (ZOA and NU) screened the first 60 records to establish the quality of screening at this stage and ascertain that the level of agreement and discrepancies were addressed through mutual consensus among the reviewers. Additional suggestions and amendments to the search teams and relevant keywords were made. There was complete agreement on the relevance of selected studies by RAE, ZA and NU.

### Inclusion criteria

Selected studies were assessed against the following inclusion criteria: (i) Peer-reviewed English articles; (ii) Population of patients prescribed antibiotics aged 18 years and over; (iii) Studies describing the AMS intervention in acute care settings; (iv) Outcomes of AMS strategies, measures, metrics before and during the COVID-19 pandemic; (v) Primary studies; and (vi) Published between 2000 and 2021. The included study designs were observational (retrospective or prospective case–control, case series non-interventional, cross-sectional, cohort) and interventional (quasi-experimental, randomised controlled trials) studies (Table 1).

### Exclusion criteria

Any study that did not fulfil the criteria for inclusion, studies unrelated to review objectives, abstract-only papers, non-human subject studies, literature and systematic review studies were excluded from this study.

### Data sources and search methods

An electronic search of International Pharmaceutical Abstracts, MEDLINE (via PubMed), CINAHL, PsychINFO, SCOPUS, Cochrane Library, Web of Science and Google Scholar [17]. Choices of databases to be searched were based on insights from the method’s section-related reviews. The search was restricted to articles published from January 2000 to March 2021 (For more details, see Supplementary Table S3). The AMS strategies and metrics identified within the MEDLINE database through the MeSH term “antimicrobial stewardship” was employed as search terms for AMS intervention. Antibiotic use before and during the COVID-19 pandemic was employed as the search term. Settings were specified as acute care settings, AND/OR were used to combine search terms (Table 2). The “snowballing” strategy, going through the reference list of all included studies to obtain further relevant studies, was also employed.

**Table 2:** The systematic literature review of search strategies

**Search Strategy**
1. Antimicrobial resistance OR antibiotic management OR acute care settings OR hospitals
2. Antimicrobial stewardship OR antimicrobial utilisation OR antimicrobial use OR antimicrobial stewardship strategies OR antibiotic metrics OR antimicrobial stewardship intervention OR antimicrobial stewardship outcomes OR antibiotic use
3. COVID19 OR coronavirus OR SARS CoV2 OR severe acute respiratory infection OR pandemic
4. 1 AND 2 AND 3
5. Limit 18–65 to yr. = ‘2000–2021’ = lang: ‘English’

(a) *COVID-19* Coronavirus

(b) *SARS CoV2* Severe Acute Respiratory Syndrome Coronavirus 2

### Quality assessment of included studies

The latest version of the Mixed Method Appraisal Tool (MMAT) was used to evaluate the quality of the included studies. Version 2018 of the MMAT was subject to content validity and usefulness [18]. Following a literature search of the databases and eligibility screening, the final included studies were independently reviewed to ensure the quality assessment's accuracy, validity and reliability. The three authors (RAE, NA and ZA) critically appraised all the included studies independently, and then the results were discussed (for more details about the quality of studies, see Supplementary Table S4).

### Data extraction and analysis/synthesis

Data extraction forms were created by the primary reviewer (RAE). It included the author's last name, year of study, country, study design, the AMS intervention strategies, AMS outcome measures, and quality of study analysis (Supplementary Figure S1). Three studies were initially piloted to test the form. RAE extracted the data from these three studies into the data extraction tool, and any discrepancies in the extracted data were discussed with the other authors. Data obtained were grouped and summarised using narrative synthesis into two groups: BP and DP (Table 3). RAE extracted the data for the included studies. In order to maintain the reliability and validity of the data extraction, another author (ABA) independently extracted the data from the included studies into data extraction form. Discrepancies in the extracted data were documented and resolved by discussion or adjudication with a third author (ZA). Meta-analysis could not be performed because of the heterogeneity of the included studies.

**Table 3:** Characteristics of Included studies before and during the COVID-19 pandemic

**Study**	**Country**	**Study type**	**AMS strategies**	**AMS Measures/Metrics**
Trivedi (2013) [19]	United States	Cross-sectional study	- AMS core strategies included formulary restriction, antibiotic review, automatic stop orders, preauthorisation, and prospective review with feedback - AMS supplemental strategies included education, dose optimisation, dose adjustments, guidelines and clinical pathways, parenteral-to-oral switch, streamlining de-escalation, and antimicrobial order forms	- Outcomes measured included antimicrobial resistance patterns (39%), antimicrobial utilisation (36%), antimicrobial costs (35%), Clostridium Difficile infection rates (32%), adverse effects (22%), 17% reported monitoring DDD and 13% reported monitoring DOT - For a positive trend in outcomes data since the initiation of the ASP, including improved antimicrobial use (74%), decreased antimicrobial costs (63%), improved antimicrobial susceptibility patterns (47%), and 38% used computer software to interface with electronic records facilitated AMS
Kallen (2017) [20]	Netherlands	Randomised clinical trial	- Data extraction and feedback on the overall antibiotic use - Point Prevalence Study of the European Centre for Disease Prevention and Control (PPS-ECDC) was conducted to provide feedback on validated Quality Indicators (QIs) for appropriate antibiotic use (PPS-QI), such as IV-to-oral switch projects (43%) and projects focusing on appropriate treatment for patients with pneumonia (21%) or the appropriate use of restricted antibiotics (19%)	Primary outcome The geometric mean LOS was 9.5 days (95% CI 8.9–10.1, *N* = 4245 patients) at baseline versus 8.7 days (95% CI 8.1–9.2, *N* = 4195 patients) after intervention while adjusting for dependencies within clusters and potential confounders. After adjusting for the secular trend, the estimated decrease in geometric mean LOS was 0.5 days: 9.5 days (95% CI 8.9–10.1, *N* = 4245 patients) at baseline versus 9.0 days after intervention (95% CI 8.5–9.6); *P* < 0.001, *N* = 4195 patients
				Secondary outcomes DOT per 100 admissions decreased from 1320 (95% CI 1253–1387, *N* = 4245 patients) at baseline to 1185 (95% CI 1119–1252, *N* = 4195 patients) after the intervention (*P* < 0.001). Similar trends were found for days of IV antibiotics. A larger decrease was found for restricted DOT per 100 admissions (*P* < 0.001). The percentage of patients admitted to the ICU was lower after the intervention (4.8%, *N* = 201 patients) compared with a baseline (5.9%, *N* = 251 patients)
Tamma (2021) [21]	United States	Prospective study	Implementation webinars of AMS, antibiotic guidelines, antibiotic time-out, clinical rounds, and antibiotic user guides, identify antibiotic safety and adverse events, antibiotic review, use innovative strategy of the four moments of antibiotic decision-making framework including: make the diagnosis, cultures, and empiric therapy, stop, narrow, change to oral antibiotics and duration	Primary outcome (Unit-Level Antibiotic Use Data): - Comparing January–February with November–December 2018, antibiotic use decreased from 900.7 to 870.4 DOT per 1000 PD (− 30.3 DOTs; 95%CI, − 52.6 to − 8.0 DOT; *P* = .008) - Fluoroquinolone use decreased from 105.0 to 84.6 DOT per 1000 PD across all units between January–February and November–December (− 20.4 DOT; 95%CI, − 25.4 to − 15.5 DOT; *P* = .009)
				Secondary outcome (C difficile identification): - The number of hospital-onset C difficile Laboratory-identified events per 10 000 PD across the Safety Program cohort was 6.3 for quarter 1, 5.3 for quarter 2, 6.0 for quarter 3, and 5.1 for quarter 4 in the 2018 calendar year in the participating units. The incidence rate of hospital-onset C difficile Laboratory-identified events decreased from quarter 1 to quarter 4 by 19.5% (95%CI, − 33.5%to − 2.4%, *P* = .03)
Surat (2021) [22]	Germany	Retrospective study	- AMS multidisciplinary committee and regular ward rounds - Formulary restriction of specific antibiotics (e.g., tigecycline and colistin) - Creation of selective antibiotic resistogram profiles - Electronic access to antimicrobial prescribing guidelines, and mobile applications - Introduction of both surveillance data on AMR and antibiotic consumption rate - In accordance with the current effective clinical practice guidelines for antimicrobial prophylaxis, the standard prophylactic regime changed from cefuroxime to cefazolin (depending on the procedure, it may differ) - Further targets involved following antibiotic groups: meropenem, which AMS strived to reduce its usage, and fluoroquinolones, which involved drastic change in hospital’s general antibiotic policy	Primary outcome (The primary endpoint was defined as the total DOT for intraabdominal infections): An overall reduction in the total days on antibiotic therapy (ABT) from a mean of 6.1 days to 4.8 days (*p* = 0.02) was noted in the antimicrobial stewardship program (ASP) period, decreasing the days of therapy per 100 patient days (DOT/100PD) from 47.0 to 42.2 (*p* = 0.035)
				Secondary outcome (The secondary endpoints included the appropriateness (indication and documentation) of the postoperative antibiotic therapy (PAT), the empiric selection of antibiotics and the frequency of antibiotic changes): - The rate of patients receiving postoperative antibiotic therapy decreased from 56.8% to 45.2% (*p* = 0.002) in the ASP period - A trend of change in the duration of postoperative antibiotic therapy from 8.1 to 7.2 days (*p* = 0.08) was observed - The individual assessments of postoperative therapy revealed significantly less inappropriate (no indication) postoperative antibiotic therapy, shortened treatment durations (not significant) and an influence on the choice of antibiotics, with the use of more narrow-spectrum antibiotics
Weston (2012) [23]	United States	Cross-sectional study	- Antibiotic restriction, by using new restriction methods, such as front-end back end, automatic stop orders, ID consult required, verbal approval required - Antibiotic guidelines and clinical pathways, antimicrobial order forms, streamlining or de-escalation, dose optimisation, parenteral-to-oral switch, and closed formulary	- Combining the results of both surveys, 31 out of 44 (70%) institutions had formal ASP in place. 13 institutions indicated on either survey that they did not have a formal ASP program. 25/38 institutions who responded to the second survey, had had an existing ASP
Mehta (2014) [24]	United States	Quasi-experimental study	Prior authorization and prospective audit with feedback	- The change from prior authorization to prospective audit with feedback was associated with a significant increase both in use of the affected antimicrobials and in overall use of all antimicrobial agents - Broad-spectrum anti-gram-negative agents that still required prior authorization during both time periods continued to decline in use after the change in ASP - The overall change in stewardship approach was associated with a significant increase in hospital LOS - During the pre-intervention period, use of broad-spectrum anti-gram-negative antibiotics was declining at a rate of − 4.00 DOT/1,000-PD per month. However, during the post-intervention period, use increased by 0.80 DOT/1,000-PD per month, indicating that the change in ASP was associated with a slope change of 4.80 DOT/1,000-PD per month (*P* < .001) - After decreasing during the 2 years before the ASP change, use of cefepime and piperacillin/tazobactam significantly increased following the transition to prospective audit with feedback by 3.21 DOT/1,000-PD per month (*P* = .003) - Overall use of all systemic antimicrobial agents significantly increased after the change in ASP method (*P* < .001) - Vancomycin use declined before the intervention but significantly increased after the intervention (*P* = .005). The use of non-audited antimicrobials significantly increased after the change in ASP methods (*P* < .001), the slope during the postintervention period continued to decline at − 1.87 DOT/1,000-PD per month - The LOT of all systemic antimicrobials declined before the intervention by − 2.30 LOT/1,000-PD per month, and, despite a significant increase in slope (*P* = .029), use continued to decrease after the intervention by − 0.33 LOT/1,000-PD per month
Moriyama, (2021) [25]	Japan	cross-sectional study	- Prospective audit and feedback protocol were observed in 23 (59.0%) hospitals when using broad-spectrum antimicrobials - Preauthorization was observed in 4 (10.3%) hospitals for using broad-spectrum antimicrobials.—Notification protocols support form was present in 37 (94.9%) for use of broad-spectrum antimicrobials	- The number of hospitals with preauthorization and notification protocols, respectively, using the investigated antibiotics were as follows: broad-spectrum antimicrobials overall 4 (10.3%) and 37 (94.9%); carbapenem 2 (5.1%) and 34 (87.2%); 3rd generation cephalosporin 0 (0%) and 0 (0%); 4th generation cephalosporin 0 (0%) and 10 (25.6%); piperacillin/tazobactam 0 (0%) and 17 (43.6%); and intravenous quinolone 3 (7.7%), and 18 (46.2%) - Regarding preauthorization and notification protocols, there were no significant differences between small/middle-sized hospitals and large hospitals - The numbers for hospitals that had intervention procedures within 7 d and 28 d, respectively, for each investigated antibiotic were as follows: broad-spectrum antimicrobials overall 17 (43.6%) and 34 (87.2%); carbapenem 16 (41.0%) and 34 (87.2%); 3rd generation cephalosporin 1 (2.6%) and 11 (28.2%); 4th generation cephalosporin 7n(17.9%) and 20 (51.3%); piperacillin/tazobactam 12 (30.8%) and 23 (59.0%); and intravenous quinolone 13 (30.8%) and 22 (56.4%). Intervention procedures to use broad-spectrum antimicrobials within 7 d were statistically more frequent in small/middle-sized hospitals than in large hospitals with findings as follows: overall, OR = 5.7, 95% CI = 1.4–23.5, *p* = 0.023; carbapenem, OR = 4.7, 95% CI = 1.1–19.1, *p* = 0.049; piperacillin/tazobactam, OR = 7.3, 95% CI = 1.3–39.9, *p* = 0.018; and intravenous quinolone, OR = 8.8, 95% CI = 1.6–48.2, *p* = 0.008
Thakkar (2021) [26]	India	Prospective study	- The pre-existing components of the hospital antimicrobial stewardship program included generation of antibiogram, formulation/ education and dissemination of antibiotic policies for surgical prophylaxis, community and hospital acquired infections and auditing antibiotics for surgical prophylaxis - Prospective audit and feedback for the restricted antimicrobials - Antibiotic restriction using the justification form	- Around 1.4% of admitted patients were put on restricted antimicrobials. The total days of therapy (DOT) were 41.5/1000 inpatient days - Unjustified use of antimicrobials was reported in 13% and recommendation of the AMS for de-escalation were accepted in 89% by the treatment team - There was no significant difference between antimicrobial DOT of the restricted antimicrobials between 2018 and 2019 - The colistin susceptibility rates remained stable compared to the previous years
Panditrao (2021) [27]	India	Quasi-experimental study	- Baseline Phase: from April–June 2017 Routine prospective audit and feedback was undertaken - Intervention Phase: from July–December 2017 The following interventions were added: Timeout, Correction of doses, continued education for rational use of antimicrobials, Care bundle approach for prevention of hospital-acquired infections (HAIs)	- There was a reduction in the cumulative DDD/1000 PD for all antimicrobials in the intervention phase compared with baseline (baseline phase 1326.3 DDD/1000PD vs. intervention phase 1313.5 DDD/1000PD) - There was no change in the average number of antimicrobials per individual patient stay in the hospital between the baseline and intervention phases; *P* = 0.59) - DOT/1000PD declined from 1112.3 in the baseline phase to 1048.6 days in the intervention phase, while LOT/1000 PD changed from 956.0 in the baseline phase to 936.3 during the intervention phase - There was a decrease in DDD/1000 PD for antimicrobials such as piperacillin/tazobactam, imipenem, meropenem, clindamycin, levofloxacin, and amikacin, while there was an increase in DDD/1000 PD of vancomycin, colistin, cefoperazone/sulbactam, metronidazole and teicoplanin - There was a decrease in percentage of carbapenem use in the intervention phase compared with the baseline phase (26.3% vs. 20.9%), whereas there was an increase in the use of polymyxins, particularly colistin (11.1% vs. 6.2%) and glycopeptides (vancomycin and teicoplanin) (12.3% vs 11.0%)
Ababneh (2020) [28]	Jordan	Cross-sectional study	This study quantified antimicrobial use in inpatient settings as part of antimicrobial stewardship program surveillance	- In terms of DDDs, carbapenems (ertapenem, meropenem, imipenem) were the most commonly used agents in a total of 28.0 DDD/100 admissions, followed by glycopeptides (vancomycin, teicoplanin) in a total of 26.8 DDD/100 admissions, piperacillin-tazobactam with 20.5 DDD/100 admissions and ceftriaxone with 14.2 DDD/100 admissions, fluoroquinolones (ciprofloxacin and levofloxacin) in a total of 11.2 DDD/100 admissions - The highest prescription rate of antibiotics was in the internal medicine wards (49.8 DDD/100 admissions), followed by surgery wards (33.2 DDD/100 admissions), intensive care unit (20.6 DDD/100 admissions), paediatrics (10.5DDD/100 admissions), oncology (10.4DDD/100 admissions) - Regarding DOTs, piperacillin-tazobactam was the most commonly used agent (27.6 DOT/100 admissions), followed by carbapenems (27.2 DOT/100 admissions), glycopeptides (24.7 DOT/100 admissions), fluoroquinolones (12.4 DOT.100 admissions), and cefazolin (11.4 DOT/100 admissions)
Spernovasilis (2021) [29]	Greece	Cross-sectional study	- Prospective audit and feedback strategy, along with a case-based education of treating doctors - Antibiotic review after 24 h, 72 h and 7 days	- Doctors believed that the prospective audit and feedback ASP strategy is more effective and educational than the preauthorization ASP strategy (70.3% and 77.7%, respectively) - Most respondents (90.6%) agreed that the implementation of an ASP improves the patients’ outcome compared to the absence of such a programme - Less than 25% of participants agreed that the prospective audit and feedback strategy of the current ASP should change - More than 80% of respondents agreed that in-person consultation is the preferred practice for the ASP and education
Ashiru-Oredope (2021) [30]	United Kingdom	Cross sectional study	- Audits and Regular surveillance of antimicrobial use/ - Point Prevalence Surveys - Quality improvement initiatives - Education, AMS meetings, multidisciplinary team and ward rounds - Writing non-COVID-19 guidelines - IV to oral switches - AMS surveillance activities - Technology (virtual meetings, virtual platforms, remote working and ward rounds) - Introduction of novel biomarkers (e.g., Procalcitonin) for differentiating viral and bacterial infections - The use of hospital electronic prescribing systems facilitated - AMS activities by antimicrobial pharmacists; allowing them to target their activities, for example, identification of patients receiving excessive durations of antibiotics - Infection prevention control - Clinics/out-patient consults and Outpatient parenteral antibiotic therapy (OPAT) - Changing current inpatient processes such as COVID-19 patients receiving a senior review more quickly - Prescribing indicators/targets reporting, and Antibiotic Review Kit (ARK) - AMS committee meeting (formal or informal)	- From qualitative open questions: respondents highlighted core AMS work e.g., reviewing and writing non-COVID-19 guidelines as being the most affected - Respondents were concerned about increased antibiotic use, delayed IV to oral switches (IVOST), and prolonged antibiotic durations - The respondents also were concerned that cases of Clostridioides difficile Infection (CDI) were rising in some hospitals - Stock shortages were also identified as difficult to manage due to overwhelmed supply chains for antibiotics, antivirals and in some cases personal protective equipment (PPE) - Positive COVID-19 outcomes included: technology being increasingly used as a tool to facilitate stewardship, e.g., virtual meetings and ward rounds - Another positive outcome was the increased introduction of novel biomarkers (e.g., procalcitonin) for differentiating viral and bacterial infections - The use of hospital electronic prescribing systems facilitated AMS activities by antimicrobial pharmacists - There has also been a positive increase in the multidisciplinary team - Increased awareness of antimicrobial guidelines and improvements seen in infection prevention
Williams (2021) [31]	United Kingdom	Retrospective study	- Seventy-three (33%) patients in the negative PCT group were on antibiotics 48 h following diagnosis of COVID-19 compared with 126 (84%) patients in the positive PCT group (*P* < 0.001), suggesting good compliance with the guideline	Primary outcome: - Patients in the negative PCT group received significantly fewer DDDs of antibiotics (both total and per alive day) compared with patients in the positive PCT group (median DDD 3.0 vs 6.8; *P* < 0.001) - A significant relationship between PCT and total DDDs remained after accounting for confounders; on average, a patient with PCT > 0.25 ng/mL had almost three-fold more DDDs of antibiotics compared with patients with PCT 0.25 ng/mL [coefficient 2.72, 95% confidence interval (CI) 2.03e3.62; *P* < 0.001]
				Secondary outcomes: - Sixty-two (28%) patients in the negative PCT group died compared with 54 (36%) patients in the positive PCT group (P.0.021), and 19 (9%) patients in the negative PCT group were admitted to the ICU compared with 28 (19%) patients in the positive PCT group (P.0.007) - Meropenem was the only carbapenem used in the study population. With specific reference to meropenem consumption, positive PCT was associated with a three-fold increase in the odds of receiving any meropenem during the course of hospital admission (odds ratio 3.16, 95% CI 1.50e6.65; *P* = 0.002)

a) *AMS* Antimicrobial Stewardship, *DDD* Defined Daily Doses, *DOT* Days of Therapy, *ASP* Antimicrobial Stewardship Program, AMR Antimicrobial Resistance

b) *PPS-ECDC* Point Prevalence Study of the European Centre for Disease Prevention and Control, *QIs* Quality Indicators

c) *PPS****-****QI* Point Prevalence Surveys, *َLOS* Length of Stay, *ICU* Intensive Care Unit, *ABT* Antibiotic Therapy

d) *PAT* Postoperative Antibiotic Therapy, *LOT* Length of Therapy, *HAIs* Hospital-Acquired Infections, *COVID-19* Coronavirus

e) *IVOST* IV to oral switches, *CDI* Clostridioides Difficile Infection, *PPE* Personal Protective Equipment

f) *OPAT* Outpatient Parenteral Antibiotic Therapy, *ARK* Antibiotic Review Kit, *PCT* Procalcitonin, *CI* Confidence Interval

^*^Though this study was published during the COVID-19 pandemic, it was conducted before the pandemic and the AMS was implemented BP

The following data were extracted for all included articles (Table 3):

- author of study;
- year of study (before or during the COVID-19 pandemic);
- country of study;
- study design;
- antimicrobial stewardship strategies;
- antimicrobial stewardship metrics/measures and quality improvements;

## Results

**Fig. 1:**
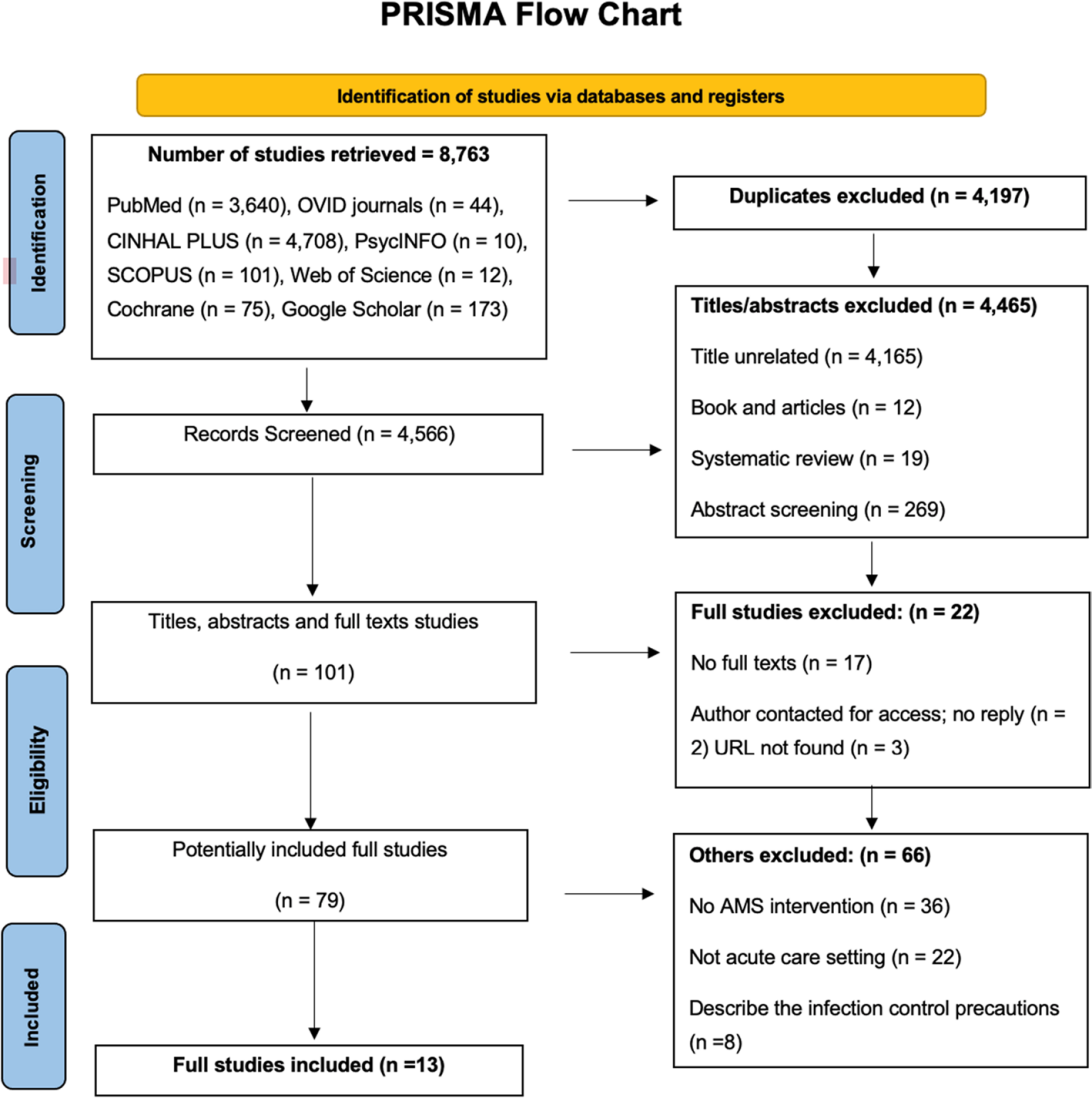
Flow chart showing the selection of eligible studies for inclusion in the systematic review

The search yielded a total of 8,763 Abstracts, which were potentially eligible for inclusion: MEDLINE (*n* = 3,640), all OVID journals (*n* = 44), CINHAL PLUS (*n* = 4,708), PsycINFO (*n* = 10), SCOPUS (*n* = 101), Web of Science (*n* = 12), Cochrane (*n* = 75), and an additional 173 records through Google Scholar. After removing duplicates, 4,566 articles remained for the title and abstract screening. One hundred and one published articles were eligible for full-text screening, of which 79 met the inclusion criteria (Fig. 1). Sixty-six articles were excluded as they had not fulfilled the inclusion criteria for the following reasons: lack of AMS intervention reported (*n* = 36), inappropriate study settings (*n* = 22), and inappropriate outcomes such as infection control precautions (*n* = 8). The final included studies were 13 (Fig. 1).

The geographical origin of the 13 studies was as follows: United States (*n* = 4) [19, 21, 23, 24], United Kingdom (*n* = 2) [30, 31], India (*n* = 2) [26, 27], Germany (*n* = 1) [22], Netherlands (*n* = 1) [20], Jordan (*n* = 1) [28], Japan (*n* = 1) [25], Greece (*n* = 1) [29]. 10 of 13 (77%) studies were conducted before the pandemic. However, only 3 of 10 (23%) studies were conducted during the COVID-19 pandemic [29–31]. The following study designs were identified: retrospective cohort (*n* = 2) [22, 31], cross-sectional (*n* = 6) [19, 23, 25, 28–30], prospective cohort (*n* = 2) [21, 26], Quasi-experimental study (*n* = 2) [24, 27], and 1 Randomized clinical trial [20]. In this review, the PHE toolkit of AMS was used as a gold standard for analysing AMS implementation. AMS strategies were categorised into AMS core & supplemental strategies according to the AMS toolkit into core and supplemental strategies [5]. Additionally, the practical guide for AMS implementation and measures was used in the analysis [8] (Table 3).

### AMS strategies before and during the COVID-19 pandemic

Strategies and interventions aimed at improving appropriate prescription of antibiotics in all acute care settings. They are considered an essential part of “antimicrobial stewardship”. According to the literature, there are many antimicrobial stewardship tools, interventions and activities (collectively termed “strategies”) that can be used to streamline and improve antimicrobial use and educate prescribers [7]. For more details about AMS strategies, see Supplementary Tables S5 and S6. In this systematic literature review, a range of AMS strategies has been classified according to the AMS implementation guidelines of the United States Infectious Disease Society of America (IDSA) and UK Public Health England AMS toolkit into core and supplemental strategies [5, 7].

Before the pandemic, regarding the core strategies, AMS Multidisciplinary Team was found in ten studies [19–28], and Prospective Audit & Feedback strategy was found in nine studies [19, 20, 22–28]. However, Antibiotic Review was noticed in seven studies [19, 21, 23, 24, 26–28]. For AMS supplemental strategies, Formulary Restriction & pre-authorisation was found in seven studies [19, 20, 22–26], Dose Optimisation strategy was found in seven studies [19, 22–24, 26–28], Streamlining/timely de-escalation of therapy strategy was found in five studies [19, 22, 23, 26, 27], Parenteral to oral conversion was found in five studies [19–21, 23, 26], and Guidelines and Clinical were found in six studies [19, 21–23, 27, 28], Antibiotic Order Form was found in two studies [19, 23], Education was found in six studies [19–23, 26, 27], Computerized Decision Support, surveillance was found in two studies [19, 23], and Laboratory Surveillance and Feedback was found in four studies [19, 22, 24, 26] (Table 4).

**Table 4:** Summary of finding about antimicrobial stewardship implementation before and during the COVID-19 pandemic

**Study**			**Antimicrobial Stewardship (AMS)**								
			**AMS Strategies**								
			**AMS Core Strategies**				**AMS Supplemental Strategies**				
	**Before-the-pandemic**	**During-the-pandemic**	**Multidisciplinary stewardship team**	**Formulary restrictions and preauthorization**	**Antibiotic Review**	**Prospective Audit and Feedback**	**Streamlining/timely de-escalation of therapy**	**Dose Optimisation**	**Parenteral to oral conversion**	**Guidelines and Clinical Pathways**	**Antibiotic Order Form**
**Trivedi (2013) **[19]	✓		✓	✓	✓	✓	✓	✓	✓	✓	✓
**Kallen (2017) **[20]	✓		✓	✓		✓			✓		
**Tamma (2021) **[21]	✓		✓		✓				✓	✓	
**Surat (2021) **[22]	✓		✓	✓		✓	✓	✓		✓	
**Weston (2012) **[23]	✓		✓	✓	✓	✓	✓	✓	✓	✓	✓
**Mehta (2014) **[24]	✓		✓	✓	✓	✓		✓			
**Moriyama (2021) **[25]	✓		✓	✓		✓					
**Thakkar (2021) **[26]	✓		✓	✓	✓	✓	✓	✓	✓		
**Panditrao (2021) **[27]	✓		✓		✓	✓	✓	✓		✓	
**Ababneh (2020) **[28]	✓		✓		✓	✓		✓		✓	
**Spernovasilis (2021) **[29]		✓	✓		✓	✓		✓		✓	
**Ashiru-Oredope (2021) **[30]		✓	✓		✓	✓	✓		✓	✓	
**Williams (2021) **[31]		✓								✓	

**Study**	**Antimicrobial Stewardship (AMS)**									
	**AMS Measure**									
	**AMS Education**	**Computerized decision support, surveillance**	**Laboratory surveillance and feedback**	**Defined Daily Dose (DDD)**	**Days of Therapy (DOT)**	**Length of Stay (LOS)**	**Cost**	**Clostridioides Difficile Infection (CDI)**	**Procalcitonin (PCT)**	**Indicators or Quality Improvement Projects**
**Trivedi (2013) **[19]	✓	✓	✓	✓	✓		✓	✓		✓
**Kallen (2017) **[20]	✓			✓	✓	✓				✓
**Tamma (2021) **[21]	✓				✓			✓		✓
**Surat (2021) **[22]	✓		✓	✓	✓	✓				✓
**Weston (2012) **[23]	✓	✓								✓
**Mehta (2014) **[24]			✓		✓	✓	✓			✓
**Moriyama (2021) **[25]							✓			
**Thakkar (2021) **[26]	✓		✓		✓					
**Panditrao (2021) **[27]	✓			✓	✓					✓
**Ababneh (2020) **[28]				✓	✓					✓
**Spernovasilis (2021) **[29]	✓		✓							
**Ashiru-Oredope (2021) **[30]	✓							✓	✓	✓
**Williams (2021) **[31]		✓	✓	✓				✓	✓	✓

*DDD* Defined Daily Doses, DOT Days of Therapy, *CDI* Clostridioides Difficile Infection, *LOS* Length of Stay, PCT Procalcitonin

**Fig. 2:**
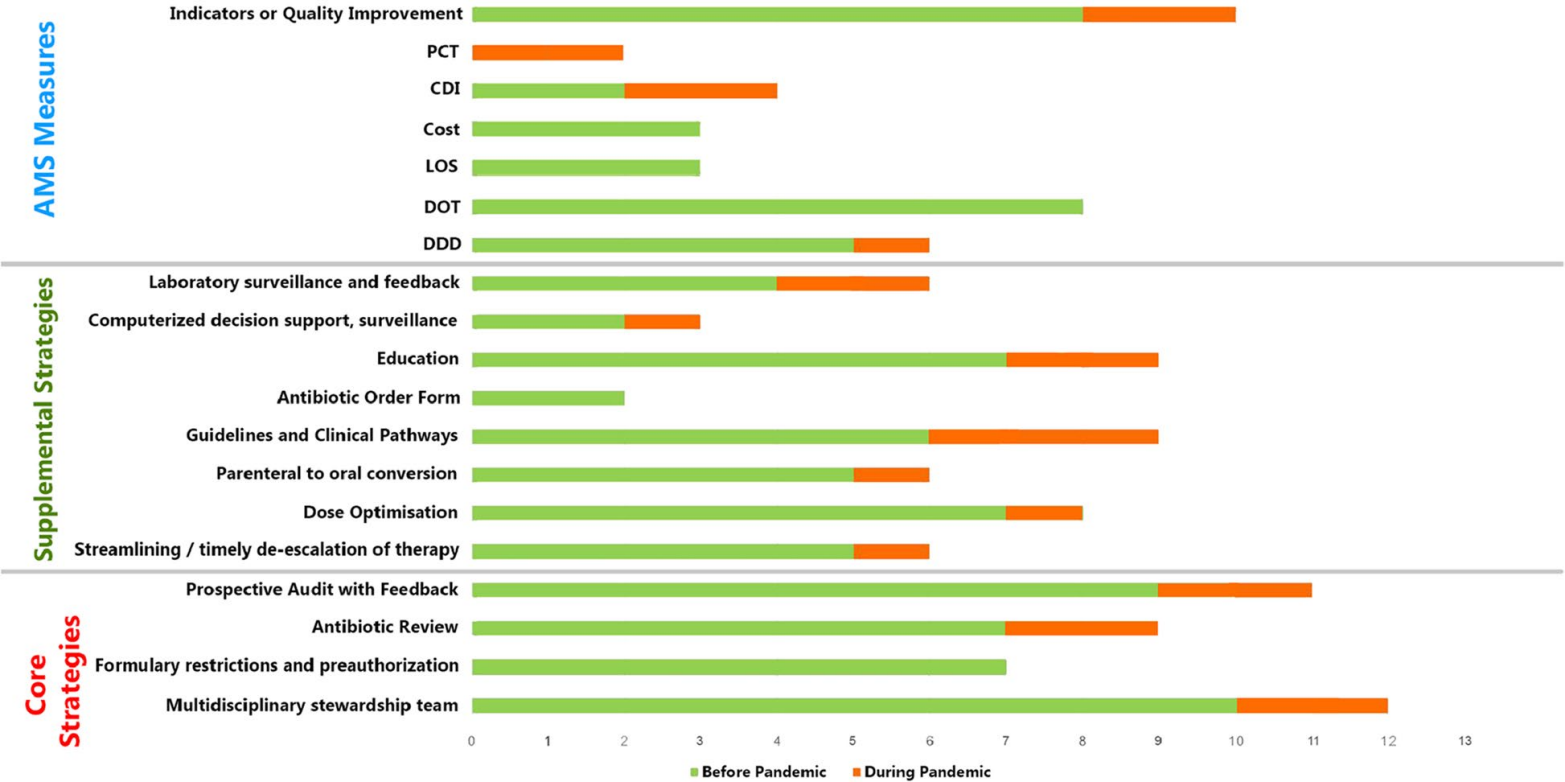
AMS before and during the COVID-19 pandemic in acute care settings (Total studies 13)

During the COVID-19 pandemic, concerning the core AMS strategies, each of AMS Multidisciplinary Team, Prospective Audit & Feedback strategy, and Antibiotic Review was found in two studies [29, 30]. For AMS supplemental strategies, Dose Optimisation strategy was found in only one study [29]. However, each Streamlining/timely de-escalation and Parenteral-to-Oral conversion was found in one study [30]. Additionally, Guidelines and Clinical Pathways were found in three studies [29–31], Education was found in two studies [29, 30], Computerized decision support and Surveillance were found in one study [31], and Laboratory surveillance and feedback found in two studies [29, 31] (Fig. 2).

### Identifying key AMS measures for improvement

Measurement of prescribing performance is essential to evaluate the impact of AMS implementation in clinical practice and its demonstrable benefits for patients. The British scientist mentioned in his Popular Lecture, “If you cannot measure it, you cannot improve it” Lord Kelvin 1824–1907 [32]. Improving antimicrobial use must be measured by Identifying the measurable elements/metrics that can be used to evaluate the outcomes of AMS. These metrics can be used for many purposes, such as quality assurance, improvement, and comparisons/benchmarking either intra-hospital or Inter-hospital. Establishing what to measure is one of the essential steps to maintain sustainability in AMS intervention [7, 33].

Measuring stewardship can be divided into four categories: antimicrobial consumption, process measures, outcome measures, and financial [34]. Before 2019, there were no reliable means for measuring antimicrobial usage or correlating usage to resistance until 2019, when the WHO promoted measurable tools that can be used worldwide to accurately reflect antimicrobial usage, such as the Defined Daily Dose (DDD) [34]. WHO defined DDD as the assumed average maintenance dose per day for the antibiotic used for its main indication in adults. To estimate the total number of days of antimicrobial therapy, healthcare personnel divide the total grams of each antimicrobial used for a given period by the WHO-defined DDD for the individual antimicrobials. Because DDD is a standardised unit of measure, it allows comparisons with antimicrobial usage in other hospitals and countries [35]. Each hospital should select suitable measures/metrics that maintain the effective implementation of the AMS. It is important to be aware of each metrics' advantages and disadvantages to maintain a proper selection. For more details about AMS outcome measures and metrics, see Supplementary Tables S8 and S9.

Before the COVID-19 pandemic, DDD was noticed in five studies [19, 20, 22, 27, 28], Days of Therapy (DOT) was found in eight studies [19–22, 24, 26–28], and Length of Stay (LOS) was found in three studies [20, 22, 24], and Cost was found in three studies [19, 24, 25], and CDI was found in two studies [19, 21] However Indicators or Quality Improvement was found in eight studies [19–24, 27, 28].

During the COVID-19 pandemic, DDD was found in only one study [31], – Clostridioides Difficile Infection (CDI) was found in two studies [30, 31], and Procalcitonin (PCT) was found in one study [31]. Indicators or Quality Improvement was found in two studies [30, 31] (Table 4) (Fig. 2).

## Discussion

This systematic review analysed data from over 63,921 patients who received antibiotics in acute care settings between 2000 and 2021. The goal was to explore strategies and measures for implementing antimicrobial stewardship (AMS). It was found that overuse and irrational use of antimicrobials is a significant problem for healthcare, which can lead to negative impacts on patient safety, the emergence of antibiotic resistance, and increased economic burden [36, 37]. The majority of respiratory tract infections, particularly Upper Respiratory Tract Infections (URTIs), are caused by viruses but are often treated with antimicrobials [38]. There is a lack of strong evidence supporting AMS implementation, which has led to confusion and disagreement about their effectiveness. This high antimicrobial consumption in COVID-19 patients was initiated after early reports from China revealed that 50% of patients died from secondary bacterial infection [39, 40]. A range of stewardship interventions has been reviewed in the IDSA guidelines [7]. When establishing a new stewardship program, it is best to start with the core strategies and focus on achieving and maintaining them before adding some supplemental strategies. A list of the Antimicrobial Stewardship Toolkit is shown in Table 4 and Fig. 2. In the published literature, effective AMS strategies should be able to decrease antimicrobial exposure, decrease costs, and improve clinical outcomes [24].

### AMS core strategies

Two core ASP strategies have emerged: front-end strategies, which involve an approval process for making antimicrobials available (formulary restrictions and pre-authorization), and back-end strategies, which involve reviewing antimicrobial use after therapy has been initiated (prospective audit with intervention and feedback). A review of these strategies found that back-end strategies, although more labour-intensive, are more widely practised, more easily accepted by clinicians, and provide more educational opportunities, leading to a more sustained impact on improving antimicrobial prescribing quality [8]. The front-end strategy used BP in 54% of studies, while the back-end strategy was used in 85% of all studies and two studies DP [29, 30].

### AMS multidisciplinary team

A multidisciplinary AMS team was found in most of the included studies, 92%. It was considered one of the key components of the structure and governance of the AMS. It consists of a core membership of an infectious disease physician (or lead doctor or physician champion), a clinical microbiologist, and a clinical pharmacist with expertise in infection (Supplementary Figure S2). Other members could be specialist nurses, for example, infection prevention or stewardship nurses, quality improvement /risk management/patient safety managers, and clinicians interested in infection. The multidisciplinary AMS team should perform a gap analysis of antimicrobial use at the facility to identify priority areas for improvement and set up a plan for AMS implementation and measurement [8]. Before the pandemic, one of the studies conducted across the United States (US) hospitals found that proper communication with the multidisciplinary AMS team was key for successful AMS implementation. For example, provide a forum for participants to ask the AMS team questions about project logistics, implementation strategies, and clinical management strategies and to share local successes and challenges. Project email addresses and designated, external site-specific quality improvement experts are also available to all participants at each site [21]. Interestingly, in 2022, there was a study conducted in Lebanon. It was the first study in Lebanon to examine the impact of the implementation of the post-prescription review and feedback (PPRF) AMS program with an infectious disease (ID) physician-driven strategy of AMS. In the intervention period of this program, there was a significant reduction in DOT, type of illness treated, types of antimicrobials in use and an indirect decrease in the length of hospital stay. Though the acceptance of the AMS multidisciplinary team recommendations were 88%, which was higher than in prior studies that typically noted an acceptance rate of 60–70%, COVID-19 was one of the limitations of this study. This is due to shortages of providers, which affected the ease of education of the treatment teams DP [41]. During the COVID-19 pandemic, a study conducted in the United Kingdom (UK) aimed to measure the COVID-19 impact on national antimicrobial stewardship (AMS) activities. There has also been a positive increase in multidisciplinary work where pharmacist contributions have been welcomed. Increased awareness of antimicrobial guidelines and improvements seen in infection prevention [30].

A multidisciplinary AMS team was found in most of the included studies, 92%. It was considered the key components of the structure and governance of the AMS. The team typically consists of an infectious disease physician, clinical microbiologist, clinical pharmacist with expertise in infection, and other members such as specialist nurses and quality improvement/patient safety managers [8]. The multidisciplinary AMS team was responsible for analysing antimicrobial use at the facility and developing a plan for AMS implementation and measurement. Communication with the AMS team was found to be important for successful AMS implementation in one study conducted in the United States [19].

### Formulary restrictions and pre-authorization

The study was conducted in Pennsylvania and compared the change from the pre-authorisation AMS strategy to the prospective audit with feedback. There was a significant increase in the use of the affected antimicrobials and the overall use of all antimicrobial agents. During the preintervention period, both total systemic antimicrobial use (− 9.75 DOT/1,000-PD per month) and broad-spectrum anti-gram-negative antimicrobial use (− 4.00 DOT/ 1,000-PD) declined [24]. Another study was conducted in Massachusetts. It aimed to study the new restriction methods, such as Front-End Back End, Automatic Stop Orders, ID Consult and Verbal Approval. It included a list of restricted antimicrobial agents (broad spectrum and later generation antimicrobials), such as New Specific Medication Restrictions: Anti-Pseudomonas, Carbapenems, Tigecycline, Vancomycin, Colistin, Daptomycin, Linezolid, Antifungals, Fluoroquinolones. The result from this study indicated that Daptomycin and Linezolid were the most frequently restricted antimicrobials [23]. An interesting study conducted in India evaluating the use of the justification form to prescribe restricted antimicrobials, such as colistin, polymyxin B, tigecycline, intravenous (IV) minocycline, IV fosfomycin, daptomycin & echinocandins (caspofungin, micafungin & anidulafungin) found that prescribing any of these antimicrobials necessitated filling an antimicrobial justification form, which was then sent to the AMS multidisciplinary committee. These forms were tallied with a daily indent list from the pharmacy of restricted antimicrobials, and any missing forms were requested to be submitted. At 48–72 h from the time of prescription, the AMS committee for review [26].

### Antibiotic review

The antibiotic review was one of the effective AMS strategies BP and DP. It was found in 69% (9 of 13) of the included studies. Antibiotic review could be conducted after 24 h (Day 1) of prescribing the antibiotics. It included a review of the doses and the possibility of an IV-to-oral switch. It also could be conducted on Day 4 to review appropriateness considering microbiological culture results or on Day 7 to review the duration of therapy [8]. We found that the antibiotic review 48–72 Hours from the time of prescription was conducted by microbiology [22] or the AMS multidisciplinary committee [26]. Interestingly, the use of the Team Antibiotic Review Form (TARF) Document by frontline prescribers was significant in decreasing antibiotic use. It was used in conjunction with antibiotic stewards for patients actively receiving antibiotics to facilitate discussions about appropriate antibiotic prescribing using the Four Moments framework; A) Make the diagnosis; B) Cultures and Empiric Therapy; C) Stop, Narrow; D) Change to Oral antibiotics; E) Duration. The use of promotional and attractive materials to promote the Four Moments of Antibiotic Decision-Making, such as posters, pocket cards, and screen savers, to advertise the Four Moments Framework. Antibiotic use was decreased by 30.3 DOT per 1000 PD (95% CI, − 52.6 to − 8.0 DOT; *P* = 0.008). Additionally, the incidence rate of hospital-onset C difficile laboratory-identified events decreased by 19.5% (95% CI, − 33.5% to − 2.4%; *P* = 0.03) [21].

Interestingly, in the study conducted in the United Kingdom (UK), 58 UK acute hospital organisations expressed an interest in participating. In England, the Department of Health's guidance Start Smart—Then Focus required prescribers to review and revise antibiotic prescriptions every 48–72 h.12. In the USA, the analogous term antibiotic timeouts is used. Still, revised Centres for Disease Control and Prevention guidance in 2019 prioritised pharmacist-led audits and feedback to prescribers. This study aimed to evaluate a multifaceted behaviour change intervention, i.e., the Antibiotic Review Kit (ARK), designed to reduce antibiotic use among adult acute general medical inpatients by increasing appropriate decisions to stop antibiotics at clinical review. It focused on decisions to stop rather than decisions to start antibiotics. Most AMS champions were microbiologists. There was no evidence that sites that achieved greater reductions in antibiotic DDDs per admission had larger increases in mortality than did sites with smaller reductions in antibiotic DDDs per admission. Interestingly, a study published in 2022 in the UK investigating the antibiotic review kit intervention resulted in sustained reductions in antibiotic use among adult acute general medical inpatients. The onset of the COVID-19 pandemic probably explains the weak, inconsistent intervention effects on mortality. Hospitals should use the antibiotic review kit to reduce antibiotic overuse. Despite its limitations, the final model adjusting for COVID-19, the ARK intervention resulted in mean reductions in antibiotic use of 4·8% per year but no immediate reduction [42].

### Prospective audit and feedback

Another study conducted in Greece. It was focused on the prescription of carbapenems with regard to the indication, dosage and duration of treatment, combined with the judicious use of carbapenem-sparing antibiotics whenever appropriate. The programme is based on the prospective audit and feedback strategy, along with a case-based education of treating doctors. An infectious diseases (ID) specialist and an ID fellow are being alerted by the hospital pharmacy upon prescription request for carbapenem and provide unsolicited in-person (“handshake”) consultation within 72 h for all patients for whom the treating doctors have prescribed carbapenem. The antibiotic review and ward rounds. Further ID consultation service upon request is available 7 days a week, 24 h a day, through telephone or in-person [29].

The Systematic implementation of AMS has shown promising outcomes. AMS was started by the Baseline Phase, which started from April to June 2017. It included a routine prospective audit, and feedback was undertaken. Followed by the Intervention Phase, which started from July–December 2017. In this phase, the following interventions were added: Timeout, Correction of doses, continued education for rational use of antimicrobials, and Care bundle approach for prevention of hospital-acquired infections (HAIs). During various interventions. 89 queries/suggestions were made during the baseline phase for 49 (52.1%) of 94 patients, while 196 queries/suggestions were made during the intervention phase for 94 (38.7%) of 243 patients. In both phases, the average number of queries raised was 2 per patient. Queries for de-escalation saw an increase in the intervention phase. This approach could be used in hospitals with limited resources in developing countries and show some benefits of such interventional strategies in resource-limited settings [27].

### AMS supplemental strategies

The Streamlining/timely de-escalation of therapy strategy was found in five studies BP and only one study DP [30]. This strategy was implemented with an antimicrobial timeout of 48 h. It consists of re-evaluating the patients’ empirical and/or definitive antimicrobial regimen, after which the antimicrobials were either continued, escalated or de-escalated according to the patient's clinical condition. This strategy was also part of the regular prospective audit and feedback, where the data-recording team kept track of the timelines and doctors in-charge regarding timeout for each patient [27].

Antibiotic de-escalation strategy in Community-Acquired Pneumonia (CAP) was one of the AMS activities that were significantly affected by the COVID-19 pandemic [30] (Table 4) (Fig. 2).

Both dose optimisation/antibiotic dose adjustment and parenteral-to-oral conversion protocols showed significant outcomes with P-values of 0.03 and 0.04, respectively, in the multi-centre study of California – US, which included 422 general acute care hospitals [19]. During the pandemic, dose optimisation could be used for the specific antibiotic, such as Carbapenems, which focused only on the prescription of carbapenems with regard to the indication, dosage and duration of treatment, combined with the judicious use of carbapenem-sparing antibiotics whenever appropriate. This approach was an essential part of AMS implementation DP [29]. Additionally, in the study assessing the Impact of COVID-19 on Antimicrobial Stewardship Activities/Programs among HCPs in the United Kingdom, respondents were concerned about increased antibiotic use, including increased use of broad-spectrum antibiotics, delayed parenteral-to-oral switch [30].

Guidelines and Clinical Pathways were the most used, as they were applied in 69% BP and DP. However, the organisational collaboration in applying the AMS guidelines and clinical pathways strategy was effectively implemented during the pandemic [30, 31]. In addition, adherence to the local, national, and international guideline recommendations is vital to prevent over- and inappropriate prescribing of antimicrobials. During the pandemic, we found that the availability of updated antimicrobial guidelines, such as the National Institute for Health and Care Excellence (NICE), as well as international guidelines from the WHO and the International Pharmaceutical Federation (FIP), were highly effective. The management of clinical pathways, such as pneumonia and respiratory tract infections in COVID-19 patients, should also be updated [30]. Additionally, the local or organisational clinical practice guidelines should be adapted based on the local antibiograms and resistogram in order to maintain the relevance of the antimicrobial guidelines, as recommended, which has an essential role in decreasing the inappropriate use of antibiotics and decreasing the AMR [22].

In Scotland, Concern regarding bacterial co-infection complicating SARS-CoV-2 has created a challenge for antimicrobial stewardship. Following the introduction of national antibiotic recommendations for suspected bacterial respiratory tract infections complicating COVID-19, a point prevalence survey of prescribing was conducted across acute hospitals in Scotland. Patients in designated COVID-19 units were included, and demographic, clinical and antimicrobial data were collected from 15 hospitals on a single day between 20 and 30th April 2020. Comparisons were made between SARS-CoV-2 positive and negative patients and patients in non-critical care and critical care units. Factors associated with antibiotic prescribing in SARS-CoV-2 positive patients were examined using Univariable and multivariable regression analyses. A relatively low prevalence of antibiotic prescribing in SARS-CoV-2 hospitalised patients and a low proportion of broad-spectrum antibiotics in non-critical care settings were observed, potentially reflecting national antimicrobial stewardship initiatives. Broad-spectrum antibiotic and antifungal prescribing in critical care units were observed, indicating the importance of infection prevention and control and stewardship initiatives in this setting [43].

### AMS education

Before the pandemic, AMS education using active learning activities showed promising results. For example, we found a study conducted across the United States (US) hospitals that applied educational activities and webinars that encouraged collaboration with the clinical microbiology laboratory, integrating nurses into stewardship activities and antibiotic allergies. This AMS educational program entitled ‘Building Stewardship: A Team Approach Enhancing Antibiotic Stewardship in Acute Care Hospitals’ offered by the Agency for Healthcare Research and Quality (AHRQ) safety program was highly effective, as it focused on the importance of Antimicrobial Stewardship Programs (ASPs), strategies for implementation, and operational issues, including an understanding of pharmacodynamics, business models, and electronic surveillance [23]. The AHRQ educational components were also used in another study in an innovative and easy way, such as 1-Page documents and accompanying user guides on infectious disease syndromes. The document could be used as (1) informational attractive display posters, (2) discussion points on clinical rounds, or (3) an outline for developing local guidelines [21]. However, during the pandemic, AMS education was found in only one study and showed an essential impact. There was a critical need for structured AMS education to deal effectively with any emergency/crisis [30].

Computer Decision Support & Surveillance and Antibiotic Order Form strategy was found only in two studies BP. However, only Computer decision support & surveillance was found in one study DP [30]. During the pandemic, the use of technology has a significant impact on AMS implementation. Positive outcomes of COVID-19 on AMS activities included: technology being increasingly used as a tool to facilitate stewardship, e.g., virtual meetings and ward rounds.

The use of hospital electronic prescribing systems facilitated AMS activities by antimicrobial pharmacists. There was a UK-wide decrease in audit activities undertaken by antimicrobial pharmacists. Additionally, PHE Fingertips data support the suspicion of increased ‘just in case’ prescribing of antimicrobials was decreased DP. The national surveillance database indicated a substantial increase in antibiotic prescribing (DDD/1000 admissions) in the COVID-19 period [30]. The use of integrated computerised systems was still effective in reducing AMR. Interestingly, the use of new technology ideas, such as mobile applications in updating the antimicrobial guidelines was effective, such as the Commonwealth Partnerships for Antimicrobial Stewardship (CwPAMS) App [30], antibiotic order forms, prescribing and availability of guidelines on smartphones [44].

Laboratory surveillance and feedback were found in 46% of the included studies. The surveillance of antimicrobial use and resistance has been used as a crucial part of AMS implementation, especially when accompanied by other strategies, such as antibiotic restriction, as shown in the study conducted in Germany. The formulary restriction of specific antibiotics (e.g., tigecycline and colistin), the creation of selective antibiotic resistogram profiles, the implementation and electronic access to antimicrobial prescribing guidelines, and mobile applications were used as AMS toolkit BP [22]. laboratory results and microbiology were essential data sources in AMS implementation [24]. During the Pandemic, reviewing the patient laboratory data was also an integral part of the patient’s clinical examination by the ID specialist or ID fellow. It is also accompanied by a review of the patient’s laboratory data, all prescribed antimicrobials, and a subsequent daily, rounding-based, in-person approach to feedback by the ID doctors. Additionally, it was used in AMS case-based education [29].

### AMS measures and quality improvement

As mentioned in the result section, there should be measures/metrics to properly manage AMS implementation. This could be conducted by identifying the measures that can be used to evaluate the outcome of AMS implementation to improve antibiotic use and AMS intervention strategies. These measures or metrics can be used for many purposes, such as quality assurance, improvement, comparisons, and benchmarking. Measuring AMS can be divided into four categories: antimicrobial consumption, process measures, outcome measures, and financial [45]. The AMS strategies have significant value with beneficial clinical, resistance and economic impact(s) [46] (Table 4) (Fig. 2). For more details, see Supplementary Table S7.

Monitoring trends in antimicrobial use and resistance within a hospital over several years and also identifying small changes in a single ward over a one-month period are essential to adapting empiric treatment according to local resistance trends, demonstrating changes in practice over time and identifying wards with high antimicrobial usage or use of non-policy antimicrobials and define targeted interventions required [8]. Surveillance of antimicrobial use and resistance is important either at the hospital, local, regional, and national levels, such as in the UK [47], Wales [48], Sweden [49], Australia [50], and Canada [51] and at the global level, such as WHO [52, 53] and ECDC [54].

Quality improvement and indicators were the most commonly used measures among the included studies, as found in about 83% of the included studies. However, quality improvement projects were found in two studies during the COVID-19 pandemic [30, 31]. It could be used at any stage of the antibiotic use process. The quality improvement activity assists clinicians in selecting the appropriate antibiotic, dose, duration, and route of administration to optimise clinical outcomes while minimising the selection of pathogenic organisms and the emergence of resistance. Importantly, there was an increasing linkage between ASPs and 146 hospital patient safety and quality initiatives. Interestingly, it was important to follow up and monitor results using appropriate quality improvement committees [19]. A single-centre quality improvement study with a retrospective evaluation of the impact of antimicrobial stewardship measures on optimising antibacterial use in intra-abdominal infections requiring emergency surgery was performed [22]. The use of the performance of a PPS to provide feedback on validated quality indicators (QIs) for appropriate antibiotic use (PPS-QI) demonstrated a reduction in geometric mean LOS of 0.8 days in the multicentre cluster-randomized clinical trial to improve antibiotic use and reduce the length of stay in hospitals in the Netherlands [20]. Quality improvement activities, such as national quality improvement schemes, were one of the AMS measures that were negatively impacted by the COVID-19 pandemic [30]. It could also be used to measure the improvement of AMS activities, such as the use of PCT-based guidelines as a useful tool for rationalising the use of antibiotics in patients with COVID-19 [31]. The presence of ongoing AMS quality indicators is one of the essential factors in maintaining preparedness for any emergency or crisis, especially at the national level [54]. During the Pandemic, an interesting study was conducted at Sheffield Teaching Hospitals NHS Foundation Trust (STHNFT). The aim of this study was to evaluate the effectiveness of the implemented guideline, which recommended that antibiotics can be withheld in patients with COVID-19 with PCT < 0.25 ng/mL unless felt necessary by a senior clinician. Additionally, the PCT in an electronic ‘COVID order set’ facilitated AMS measures and surveillance was included. This study found that a PCT-based guideline can be a useful tool for rationalising the use of antibiotics in patients with COVID-19 [31].

Both LOS and Cost were found in three studies, only BP. The use of LOS had several advantages: it was easy to measure, could be applied to all admitted patients, reflected the recovery time of hospitalised patients and drove hospital costs [20]. LOS was used to examine the antimicrobial use and length of stay (LOS) before and after a change in AMS approach at the Hospital of the University of Pennsylvania, a 776-bed tertiary care academic medical centre in Philadelphia and showed a significant increase after the change in AMS strategy from Pre-authorization and Prospective Audit with Feedback [24]. Interestingly, when prior to authorisation, AMS strategy was conducted in costly antibiotics, such as including aztreonam, ceftazidime, daptomycin, levofloxacin, linezolid, and meropenem) and showed a promising outcome in decreasing the LOS and cost. LOS is an important factor in healthcare cost analysis. Based on the national health insurance claims database and specific health check-ups in Japan, the importance of appropriate use of antibiotics and AMS implementation was paramount [25].

Before 2019, there were no reliable means for measuring antimicrobial usage. The WHO promoted measurable tools, such as the defined daily dose (DDD) and Day of Therapy (DOT), to allow comparisons for antimicrobial usage among hospitals and countries [33, 55]. In the included studies, the DDD and DOT are the most common AMS measures, as it was used in 53% of BP and 28% of DP. Significantly, we found another study promoted the use of KPIs, such as the AMR local indicators—produced by the UKHSA among the National Health Service (NHS) hospitals in England, and it showed a significant outcome in AMS and provided a comparative measure for the antibiotic prescribing among different periods DP [30, 56].

On the other hand, the CDI rate was used in measuring the outcome of AMS implementation [19]. It was found that a reduction in antibiotic use and hospital-onset CDI rates was an outcome of implementing the Agency for Healthcare Research and Quality Safety Program across US hospitals [21]. During the pandemic, there was a concern about increasing CDI rates as a result of the COVID-19 pandemic across all National Health Service (NHS) acute trusts in England [30]. Interestingly, data on CDI was collected as a contribution to AMS activities DP [31].

A study published in Cambridge University Press aimed to develop and implement antibiotic stewardship activities in urgent care targeting non–antibiotic-appropriate acute respiratory tract infections (ARIs). The AMS activities were started in fiscal 2020 and included measure development, comparative feedback, and clinician and patient education. This study measured antibiotic prescribing in fiscal years 2019, 2020, and 2021 for the stewardship targets, potential diagnosis-shifting visits, and overall. Additionally, it collected patient satisfaction data for ARI visits. The antibiotic prescribing rate decreased for stewardship-measure visits from 34% in FY19 to 12% in FY21. Although AMS was affected by the COVID-19 pandemic, an ambulatory antimicrobial stewardship program that focused on improving non–antibiotic-appropriate ARI prescribing was associated with decreased prescribing for (1) the stewardship target, (2) a diagnosis-shifting measure, and (3) overall antibiotic [57]. The first step to improving the current situation is to measure how medicines are used and this forms the basis of advocacy for change [58]. Clinical pharmacist has a critical role in AMS, and can be effective in implemented sustainable change [59].

### Limitations of the systematic review

Searching only published databases could have resulted in missing some potentially relevant but unpublished studies from the review. Secondly, limiting studies to being published in English could have resulted in missing essential studies published in other languages.

### Limitations of the evidence

To the knowledge of the authors, this is the first systematic review to assess the AMS implementation of BP and DP. However, there are insufficient studies using AMS strategies and measures. The authors did their best to compare the AMS strategies and measures, but variations in their use affected the comparability of findings across studies.

### Comparison with existing literature

A few reviews have assessed AMS in hospitalised patients. However, none of the reviewers has focused on the core and supplemental AMS strategies, nor the AMS measures in secondary care and acute care settings BP and DP as explored in this present systematic review.

### Implications for research and practice

Few studies identified the AMS measures, the use of AMS indicators and quality improvement projects which are relevant to this systematic review. Therefore, further studies are required to provide measurable indicators for assessing AMS implementation. It will also enable the planning and evaluation of suitable AMS interventions. Secondly, further research is required to develop methods for standardised measurements for AMS implementation that will allow greater comparability of AMS outcomes and measures across studies. Lastly, there was evidence that antibiotic use is best achieved with organisational collaboration, especially during an emergency or pandemic.

## Conclusion and recommendations

This systematic literature review investigated the AMS strategies and measures used in the acute care settings BP and DP. Advocacy for AMS must continue in the post-pandemic era to assure the safety of patient care. There are so many lessons learnt from the COVID-19 pandemic. These lessons and further recommendations from this systematic review were as follows:

1. In order to set up AMS, a multidisciplinary team is one of the key components of the structure and governance of the ASP in acute care settings BP and DP.
2. When establishing a new stewardship program, it is best to start with the core strategies and focus on achieving and maintaining them before adding some of the supplemental strategies.
3. Each Hospital should select the relevant AMS intervention tools to maintain the appropriate use of antibiotics and decrease the AMR. The types of interventions selected, how to be delivered, and by whom will be determined by local resources need and available expertise.
4. A prospective Audit with Feedback and Antibiotic Review core strategies showed promising outcomes in AMS implementation DP.
5. Guidelines, & Clinical Pathways, Guidelines and Education strategies were important to maintain the successful implementation of AMS BP and DP.
6. The development of national prescribing indicators helped to promote the appropriate antibiotic use during-the-pandemic, such as the UK five-year National Action Plan 2019–2024, with ambitions to reduce UK antimicrobial use in humans by 15% by 2024.
7. Novel AMS measures, such as Procalcitonin-guided antibiotic prescribing, showed a promising effect on AMS implementation. Results showed reduced antibiotic consumption in patients with PCT 0.25 ng/mL with no increase in mortality. Further research is recommended to identify the optimal cut-off value for PCT in this setting.
8. DDD and DOT are the most common AMS measures among the other measures. There is a need to standardise AMS measures in order to provide a comparison of outcomes and planning of effective AMS implementation.
9. The use of an integrated Computerised Decision Support System and Surveillance is required to maximise the use of technical support in sustained AMS implementation and measuring, which would be beneficial in preparing for any future crisis or emergencies.

## Supplementary Information

**Additional file 1: Supplementary Table S1.** Descriptive definitions of Antimicrobial Stewardship. **Supplementary Table S2.** Rationale behind selecting each database used to conduct the systematic literature review. **Supplementary Table S3**. The systematic review of the search terms in different databases. **Supplementary Table S4.** The quality of the included studies using MAAT. **Supplementary Table S5.** Antimicrobial Stewardship Core and Supplemental Strategies. **Supplementary Table S6.** Definition of some of AMS strategies. **Supplementary Table S7.** AMS Strategies and their related outcomes. **Supplementary Table S8.** Suggested measures for antimicrobial stewardship. **Supplementary Table S9.** ASP Metrics Example. **Supplementary Figure S1.** Data extraction Form. **Supplementary Figure S2.** A multidisciplinary approach to antimicrobial stewardship implementation.

## Data Availability

All data supporting the conclusions of this article are included within the article and a list of references.

## Abbreviations

ABT: Antibiotic Therapy
AHRQ: Agency for Healthcare Research and Quality
AMS: Antimicrobial Stewardship
AMS: Antimicrobial Stewardship
ARK: Antibiotic Kit Review
ASP: Antimicrobial Stewardship Program
AMR: Antimicrobial Resistance
ARIs: Acute respiratory tract infections
BP: Before-the-pandemic
BSAC: British Society for Antimicrobial Chemotherapy
CAP: Community-Acquired Pneumonia
CBA: Controlled Before-After
CDI: Clostridioides Difficile Infection
CI: Confidence Interval
COVID-19: Coronavirus
CwPAMS: Commonwealth Partnerships for Antimicrobial Stewardship
DDD: Defined Daily Doses
DOT: Days of Therapy
DP: During-the-pandemic
ECDC: European Centre for Disease Prevention and Control
FIP: International Pharmaceutical Federation
HAIs: Hospital-Acquired Infections
HCPs: Healthcare Professionals
ICU: Intensive Care Unit
IVOST: IV to oral switches
IDSA: The Infectious Diseases Society of America
IV: Intravenous
ISRCTN: International Standard Randomised Controlled Trial Number
LOS: Length of Stay
LOT: Length of Therapy
MMAT: Mixed Method Appraisal Tool
NHS: National Health Service
NICE: National Institute for Health and Care Excellence
STHNFT: Sheffield Teaching Hospitals NHS Foundation Trust
OPAT: Outpatient Parenteral Antibiotic Therapy
PAT: Postoperative Antibiotic Therapy
PCT: Procalcitonin
PPE: Personal Protective Equipment
PPS-ECDC: Point Prevalence Study of the European Centre for Disease Prevention and Control
PPS**-**QI: Point Prevalence Surveys; LOS—Length of Stay; ICU—Intensive Care Unit; ABT—Antibiotic Therapy
PHE: Public Health England
PICOS: Population, Intervention, Comparison, Outcome, Setting
PRISMA: Preferred Reporting Items for Systematic Reviews
PPS-ECDC: Point Prevalence Study of the European Centre for Disease Prevention and Control
PPRF: Post-prescription review and feedback
QIs: Quality Indicators
RCTs: Randomized Controlled Trials
STHNFT: Sheffield Teaching Hospitals NHS Foundation Trust
SARS-CoV-2; COVID-19: Severe acute respiratory syndrome coronavirus-2
TARF: Team Antibiotic Review Form
URTIs: Upper Respiratory Tract Infections
US: United States
UK: United Kingdom
UH: University of Hertfordshire
WHO: World Health Organization
