# Supplementary material for: Antimicrobial stewardship implementation before and during the COVID-19 pandemic in the acute care settings: a systematic review
Source: BMC Public Health. 2023 Feb 10;23:309. doi: 10.1186/s12889-023-15072-5 (PMC9918338; doi:10.1186/s12889-023-15072-5)
Supplement: Supplementary file 1 — Additional file 1: Supplementary Table S1. Descriptive definitions of Antimicrobial Stewardship. Supplementary Table S2. Rationale behind selecting each database used to conduct the systematic literature review. Supplementary Table S3. The systematic review of the search terms in different databases. Supplementary Table S4. The quality of the included studies using MAAT. Supplementary Table S5. Antimicrobial Stewardship Core and Supplemental Strategies. Supplementary Table S6. Definition of some of AMS strategies. Supplementary Table S7. AMS Strategies and their related outcomes. Supplementary Table S8. Suggested measures for antimicrobial stewardship. Supplementary Table S9. ASP Metrics Example. Supplementary Figure S1. Data extraction Form. Supplementary Figure S2. A multidisciplinary approach to antimicrobial stewardship implementation. [file 12889_2023_15072_MOESM1_ESM.docx]

**
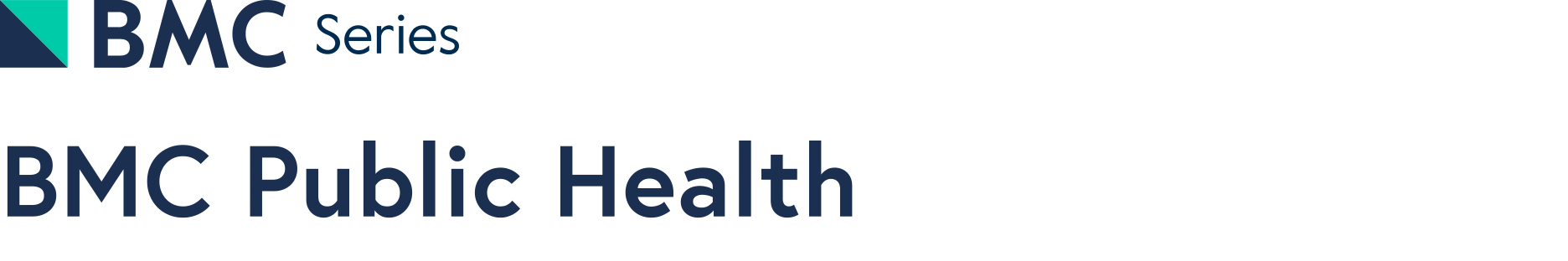
**

**Supplementary appendix**

This appendix formed part of the original submission and has been peer-reviewed. For the study of ‘**Antimicrobial Stewardship Intervention Before and During The COVID-19 Pandemic in the Acute Care Settings: A Systematic Review’**. We post it as supplied by the authors.

Supplement to: Rasha Abdelsalam Elshenawy 1*, Nkiruka Umaru1, Amal Bandar Alharbi1, Zoe Aslanpour1

1 Department of Clinical, Pharmaceutical and Biological Sciences, University of Hertfordshire School of Life and Medical Sciences, United Kingdom, AL10 9AB

*Corresponding author. Email: R.a.elshenawy@herts.ac.uk

**Table of Contents**

1. **Supplementary Tables...............................................................................**

**1.1. Supplementary Table S1: Descriptive definitions of Antimicrobial Stewardship.**

**1.2. Supplementary Table S2: Rationale behind selecting each database used to conduct the systematic literature review.**

**1.3. Supplementary Table S3: The systematic review of the search terms in different databases**

**1.4. Supplementary Table S4: The quality of the included studies using MAAT.**

**1.5. Supplementary Table S5: Antimicrobial Stewardship Core and Supplemental Strategies.**

**1.6. Supplementary Table S6: Definition of some of AMS strategies**

**1.7. Supplementary Table S7: AMS Strategies and their related outcomes**

**1.8. Supplementary Table S8: Suggested measures for antimicrobial stewardship.**

**1.9. Supplementary Table S9: ASP Metrics Example.**

**2. Supplementary Figures..........................................................................**

**2.1. Supplementary Figure S1: Data extraction Form**

**2.3. Supplementary Figure S2: A multidisciplinary approach to antimicrobial stewardship implementation**

**Supplementary Tables**

**Supplementary Table S1: Descriptive definitions of Antimicrobial Stewardship**

| **Antimicrobial Stewardship Definitions** | |
| --- | --- |
| **Antimicrobials and antimicrobial medicines** | The NICE and PHE defined the term 'antimicrobials' and 'antimicrobial medicines' as all anti-infective therapies, (antiviral, antifungal, antibacterial and antiparasitic medicines) and all formulations (oral, parenteral, and topical agents) [1]. |
| **Antimicrobial Resistance (AMR)** | The NICE and PHE defined the term 'antimicrobial resistance' as the 'loss of effectiveness of any anti-infective medicine, including antiviral, antifungal, antibacterial and antiparasitic medicines' [1]. |
| **Antimicrobial Stewardship (AMS)** | The NICE and PHE defined 'antimicrobial stewardship' as 'an organisational or healthcare-system-wide approach to promoting and monitoring judicious use of antimicrobials to preserve their future effectiveness' [1]. |
| **Antimicrobial Stewardship Program (ASP)** | ﻿The WHO defined ASP as an organizational or system-wide health-care strategy to promote the appropriate use of antimicrobials through the implementation of evidence-based interventions [2]. |

**Supplementary Table S2: Rationale behind selecting each database used to conduct the systematic literature review**

| **Database** | **Rational** |
| --- | --- |
| PubMed | Free full-text database that covers MEDLINE and EMBASE journals from life and biomedical sciences, including papers not yet indexed in MEDLINE |
| Scopus | Freely available; one of the most substantial citations and abstract database of peer-reviewed literature, including journals and conference abstracts |
| PsycINFO | This weekly updated database is considered the most significant resource in mental and behavioural sciences, including different types of literature, such as dissertation abstracts |
| CINHAL Plus | Covers a wide range of health topics including nursing, health and allied medical sciences |
| Web of Science | Consists of many databases and citations including Conference Proceedings Citation Index – Social Science & Humanities and MEDLINE |
| All Ovid journals | Includes numerous journals including health and medical journals |
| OpenGrey | Consists of grey literature including research reports, doctoral dissertations, and several conference papers |

**Supplementary Table S3: The systematic review of the search terms in different databases**

| **Database** | ***Search term*** |
| --- | --- |
| **PubMed** | *((((((((((((((((((antimicrobial stewardship[Title/Abstract]) OR (“antimicrobial utilization”[Title/Abstract])) OR (“antimicrobial use”[Title/Abstract])) OR (“antimicrobial stewardship strategies”[Title/Abstract])) OR (“antibiotic metrics”[Title/Abstract])) OR (“antimicrobial stewardship intervention”[Title/Abstract])) OR (“antimicrobial stewardship outcomes”[Title/Abstract])) OR (“antibiotic use”[Title/Abstract])) AND (“COVID19”[Title/Abstract])) OR (“coronavirus”[Title/Abstract])) OR (“SARS CoV2”[Title/Abstract])) OR (“severe acute respiratory infection”[Title/Abstract])) OR (“pandemic”[Title/Abstract])) AND (“antimicrobial resistance”[Title/Abstract])) OR (“antibiotic management”[Title/Abstract])) OR (“acute care settings”[Title/Abstract])) OR (“hospitals”[Title/Abstract])) Sorted by: best match* |
| **Scopus** | *( TITLE-ABS-KEY ( “antimicrobial stewardship” or “antimicrobial utilization” or “antimicrobial use” or “antimicrobial stewardship strategies” or “metrics” or “intervention” or “antibiotic use” and “COVID-19” or COVID19” or “coronavirus” or “SARS-CoV-2” or “severe acute respiratory infection” or “pandemic” and “antimicrobial resistance” or “antibiotic management” or “acute-care settings” or “hospitals” )  AND  TITLE-ABS-KEY ( hospital  OR  hospitalized  OR  admitted  OR  admissions  OR  "secondary care"  OR  hospitalization ) )  AND  ( LIMIT-TO ( LANGUAGE ,  "English" ) )* |
| **CINHAL PLUS** | *“Antimicrobial stewardship” OR “antimicrobial utilization” OR “antimicrobial use” OR “antimicrobial stewardship strategies” OR “antibiotic metrics” OR “antimicrobial stewardship intervention” OR “antimicrobial stewardship outcomes” OR “antibiotic use” AND “COVID19” OR “coronavirus” OR “SARS CoV2” OR “severe acute respiratory infection” OR “pandemic” AND “antimicrobial resistance” OR “antibiotic management” OR “acute care settings” OR “hospitals”* |
| **All OVID journals,**  **PsycINFO and**  **Web of Science** | *((“antimicrobial stewardship” or “antimicrobial utilization” or “antimicrobial use” or “antimicrobial stewardship strategies” or “metrics” or “intervention” “antibiotic use” and (“COVID-19” or COVID19” or “coronavirus” or “SARS-CoV-2” or “severe acute respiratory infection” or “pandemic”) and (“antimicrobial resistance” or “antibiotic management” and “acute-care settings” or “hospitals”))* |
| **OpenGrey** | *"“antimicrobial stewardship” OR “antimicrobial utilization” OR “antimicrobial use” OR “antimicrobial stewardship strategies” OR “antibiotic metrics” OR “antimicrobial stewardship intervention” OR “antimicrobial stewardship outcomes” OR “antibiotic use” AND “COVID19” OR “coronavirus” OR “SARS CoV2” OR “severe acute respiratory infection” OR “pandemic” AND “antimicrobial resistance” OR “antibiotic management” OR “acute care settings” OR “hospitals” AND admissions OR Hospital* OR hospital* OR admitted lang:"en"* |

**Supplementary Table S4: The quality of the included studies using MAAT.**

|  | **Low** | **Medium** | **High** |
| --- | --- | --- | --- |
| Trivedi et al (2013) [21] |  |  |  |
| Kallen et al (2017) [19] |  |  |  |
| Tamma et al (2021) [26] |  |  |  |
| Weston et al (2012) [18] |  |  |  |
| Surat (2021) [25] |  |  |  |
| Mehta et al (2014) [33] |  |  |  |
| Moriyama et al (2021) [32] |  |  |  |
| Thakkar et al (2021) [27] |  |  |  |
| Panditrao et al (2021) [34] |  |  |  |
| Ababneh et al (2020) [23] |  |  |  |
| Spernovasilis (2021) [22] |  |  |  |
| Ashiru-Oredope (2021) [28] |  |  |  |
| Williams (2021) [29] |  |  |  |

**Supplementary Table S5: Antimicrobial Stewardship Core and Supplemental Strategies [3]**

| **Core Strategies** | **Supplemental Strategies** |
| --- | --- |
| Formulary restrictions and pre-authorization | Streamlining / timely de-escalation of therapy |
| Prospective audit with feedback | Dose optimization |
| Multidisciplinary stewardship team | Parenteral to oral conversion |
|  | Guidelines and clinical pathways |
|  | Antimicrobial order forms |
|  | Education |
|  | Computerized decision support, surveillance |
|  | Laboratory surveillance and feedback |

**Supplementary Table S6: Definition of some of the AMS strategies [4].**

| **Intervention/**  **Strategies** | **Description** |
| --- | --- |
| **Formulary restriction** | Antibiotics may be prescribed only:   - For certain approved clinical indications - By certain physicians (i.e., infectious diseases specialists) |
| **Pre-authorisation** | Permission (from ASP team member or infectious diseases specialist) required for release of certain antibiotics. Often implemented together with formulary restriction. |
| **Prospective audit and feedback** | Case review by trained ASP team member and feedback of recommendations if reviewed antibiotics are deemed to be inappropriately prescribed. |
| **Clinical guidelines** | Treatment protocols for various infections – should be institution-specific |
| **Clinical decision support systems** | Information technology systems for improving antibiotic prescription. Requires existing electronic records and electronic prescribing system to be effective |
| **Microbiology laboratory susceptibility reporting** | Selective reporting of susceptibility profiles for positive cultures may dramatically alter prescribing patterns of physicians |

# **Supplementary Table S7: AMS Strategies and their related outcomes**

| **Strategy** | **Evidence Support Outcomes** |
| --- | --- |
| Formulary restriction | Clinical Outcome  Economic Outcome  Resistance Outcome |
| Formulary review/streamlining | Clinical Outcome |
| General antimicrobial order forms | Clinical Outcome |
| Identification of inappropriate pathogen/antimicrobial combinations (bug-drug mismatch | Resistance Outcome |
| Improved rapid diagnostics | Clinical Outcome  Economic Outcome  Resistance Outcome |
| Intravenous to oral conversion | Clinical Outcome  Economic Outcome  Resistance Outcome |
| Prescriber education | Clinical Outcome  Economic Outcome  Resistance Outcome |
| Preventing treatment of non-infectious conditions | Resistance Outcome |
| Promotion of timely and appropriate microbiologic sampling | Resistance Outcome |
| Prospective audit with intervention and feedback | Clinical Outcome  Economic Outcome  Resistance Outcome |
| Scheduled antimicrobial reassessments ("antibiotic time-outs") | Economic Outcome  Resistance Outcome |
| Strategic microbiology results reporting | Resistance Outcome |
| Surgical antibiotic prophylaxis optimization | Clinical Outcome |
| Systematic antibiotic allergy verification | Clinical Outcome |
| Targeted review of patients with Clostridium difficile infection | Clinical Outcome  Resistance Outcome |
| Targeted review of patients with bacteremia/fungemia | Clinical Outcome  Resistance Outcome |
| Targeted review of therapeutic duplication | Economic Outcome |
| Therapeutic drug monitoring (with feedback) | Clinical Outcome |

# **Supplementary Table S8: Suggested measures for antimicrobial stewardship** **[5]**

| **AMS program measures for quality improvement** |
| --- |
| **Structural indicators** |
| - Availability of multi-disciplinary antimicrobial stewardship team - Availability of guidelines for empiric treatment and surgical prophylaxis - Provision of education in the last 2 years |
| **Process measures** |
| - Amount of antibiotic in DDD/100 bed days   - Promoted antibiotics   - Restricted antibiotics - Compliance with acute empiric guidance (policy compliance) - % Appropriate de-escalation; % appropriate switch from IV to oral - Compliance with surgical prophylaxis (<60 min from incision, <24 hours and compliance with local policy) - Compliance with care “bundles” – (3-day antibiotic review bundle, ventilator-associated pneumonia, community-acquired pneumonia, sepsis) |
| **Outcome measures** |
| - C. difficile rates - Surgical Site Infection (SSI) rates - Surveillance of resistance - Mortality Rates - Treatment-related toxicity (e.g., aminoglycoside-related toxicity) - Rate of complications - Readmission within 30 days of discharge |

# **Supplementary Table S9: AMS Metrics Example [6]**

| **Metric** | **Definition** | **Example** | | | | |
| --- | --- | --- | --- | --- | --- | --- |
| Outcome Measures (Antimicrobial Utilization Measures and Antimicrobial Resistance Measures) | | | | | | |
| Grams of antimicrobials | Grams of antimicrobial based on acquisition (purchased), dispensed, or administered over a defined time | Measure the grams of antimicrobials in three different baselines (pre-, during, after wave1 and wave 2 of pandemics | | | | |
| Antimicrobial Expenditures | Antimicrobial costs can be based on dispensed or administered over a defined time  Costs can be expressed as absolute £ value, percent of total (dispensed or administered) and/or per patient-days  Antimicrobials can be tracked monthly hospital wide, for specific clinical services (e.g., ICU), classes of antimicrobials (e.g., fluoroquinolones), individual drugs (e.g., linezolid), or types of infections/indications (e.g. ventilator associated pneumonia) | For example, Pharmacy drug budget of £3,000,000 Antimicrobial acquisition costs £750,000 (25% of budget)  Cost savings (percent reduction in antimicrobial costs):  a) overall antibiotic acquisition costs  During: £750,000  Post COVID: $675,000  Absolute decrease of £75,000, equals 10% reduction  b) ICU antibiotic acquisition costs  During pandemics: £100,000 (patient days = 2000, $50/patient-day)  Pre: £75,000 (patient days = 2000, £37.50/patient-day) Absolute decrease of $25,000, equivalent to a reduction of $12.50/patient-day | | | | |
| Defined Daily Dose (DDD) | “The assumed average maintenance dose per day for a drug used for its main indication in adults” as specified by the World Health Organization (WHO). (e.g., Levofloxacin = 500mg daily)  DDD are often standardized to 1000 patient days (DDD/1000 patient days) to allow comparison of antibiotic use during pandemics and before it. | Refer to the WHO-approved Defined Daily Dose values  1 levofloxacin DDD = 0.5 g Rx: Levofloxacin 500mg po od x 7 days DDD = (0.5g dose / 0.5g DDD) x 7d = 1 DDD x 7d = 7 DDD  Rx: Levofloxacin 750mg po od x 7 days DDD = (0.75g dose / 0.5g DDD) x 7d = 1.5 DDD x 7d = 10.5 DDD Rx: Levofloxacin 750mg po q48h x 7 days DDD = (0.75g/0.5g DDD) x 4 (# days on which patient received a dose) = 6 DDD  Pre pandemics: hospital dispensed 13,000 grams of meropenem, WHO DDD for meropenem: 2 g = 6500 DDD (13,000 / 2) If 391,116 occupied bed days after pandemics then 6500 DDD / 391,116 X 1000 = 16.6 DDD / 1000 patient days | | | | |
| Days of Therapy (DOT) | The number of days that a patient receives an antimicrobial agent (regardless of dose). Any dose of an antibiotic that is received during a 24- hour period represents 1 DOT. The DOT for a given patient on multiple antibiotics will be the sum of DOT for each antibiotic that the patient is receiving. DOT is often standardized to 1000 patient days (DOT/1000 patient days) to allow comparison between hospitals or services of different sizes. | Rx: Levofloxacin 500mg po od x 7 days DOT = 1 DOT x 7d = 7 DOT  Rx: Levofloxacin 750mg po od x 7 days DOT = 1 DOT x 7d = 7 DOT  Rx: Levofloxacin 750mg po q48h x 7days = 4 DOT  Rx: Cefazolin 2 g q8h iv X 1 day = 1 DOT  Rx: Cefazolin 1 g iv X 1 dose = 1 DOT  Rx: Levofloxacin 750mg po od x 7 days + Vancomycin 1g iv q12h x 7 days:  DOT Levofloxacin = 1 DOT x 7d = 7 DOT  DOT Vancomycin = 1 DOT x 7d = 7 DOT  Total DOT = 14 DOT | | | | |
| Antimicrobial Resistance Trends | Number of patients with a specific drug-resistant organism divided by the total number of patients admitted to the ward, service, or unit of interest | Meropenem resistant Pseudomonas aeruginosa in critical care:  During the pandemics, of 500 patients admitted to critical care unit, 100 patients had meropenem resistant P. aeruginosa: 100/500 = 20%  60 patients with meropenem resistant P. aeruginosa in 2012 with 600 patients admitted to critical care unit pre pandemics: 60/600 = 10%  Therefore, the rate of meropenem-resistant P. aeruginosa was reduced from 20% in 2009 to 10% pre pandemics. | | | | |
| C. difficile Infection (CDI) rate | CDI rate per 1,000 patient days: Number of patients newly diagnosed with institution acquired CDI, divided by the number of inpatient days in that time, multiplied by 1,000  May also be expressed as the number of new CDI cases per 1000 patient admissions  For more information on the testing, management and surveillance of CDI see Annex C: Routine Practices and Additional Precautions | During pandemics: 75 cases C. difficile and 90,000 patient days in 2009 = (75/90,000) *1000 = 0.83  Before pandemics: 43 cases C. difficile and 85,000 patient days in 2011 = (43/85,000) *1000 = 0.5  Reduction in C. difficile rate = (0.83-0.5)/0.83 = 40% reduction in C. difficile rate in 2011 compared to 2009 | | | | |
| Hospital Associated Antibiotic Resistant Organism (ARO) Infection Rate | New hospital associated Methicillin Resistant Staphylococcus aureus (MRSA) bacteremia rate per 1,000 patient days or new hospital associated  Vancomycin Resistant Enterococcus (VRE) bacteremia rate per 1,000 patient days | 2 cases MRSA bacteremia  April - June  Patient days = 2100  Rate = (2/2100) *1000 = 0.95 | | | | |
| Process Measures | | | | | | |
| Interventions | Tally of the number and type of interventions made and acceptance rate | 1000 antimicrobial orders were reviewed by the stewardship team in pre pandemics and recommendations were made for 750 (75%)  The overall acceptance rate was 650/750 (87%) | | | | |
|  | Potential types of interventions are listed in the sample calculation | The types of interventions and their acceptance rates were: | | | | |
|  |  | Dose optimization  n= 152/160 (95%) | Escalation of therapy  n=45/50 (90%) | De-escalation of therapy  n=250/300 (83%) | Route change (e.g., IV to PO)  n=89/100 (89%) | Discontinuation of therapy  n=112/140 (80%) |

**Supplementary Figures**

**Supplementary Figure S1: Data extraction Form:**

1. General information

| Title of the article |  |
| --- | --- |
| First author and Date published |  |
| Country of study, and country classification |  |
| Conflict of interest |  |
| Notes: | |

1. Eligibility

| Type of the study |  |
| --- | --- |
| Population |  |
| Primary and secondary outcomes measure |  |
| Decision |  |
| Notes: | |

1. If included.

| Quality assessment score |  |
| --- | --- |
| Aim of the study |  |
| Population and location description |  |
| Design |  |
| Study period |  |
| Aim of the Study |  |
| Total number of participants |  |
| Location of the study, hospital, or department |  |
| Results   - ASM strategies, measures, metrics, Quality Improvement and KPIs detected - Strategies and measures of ASM used - HCP or stakeholders involved - Metrics of ASM - Common Strategies used before and during COVID-19 pandemics - Other results |  |
| Authors Conclusion |  |
| Strength of the study |  |
| Limitation of the study |  |
| Notes: | |

**Supplementary Figure S2: A multidisciplinary approach to implement AMS**


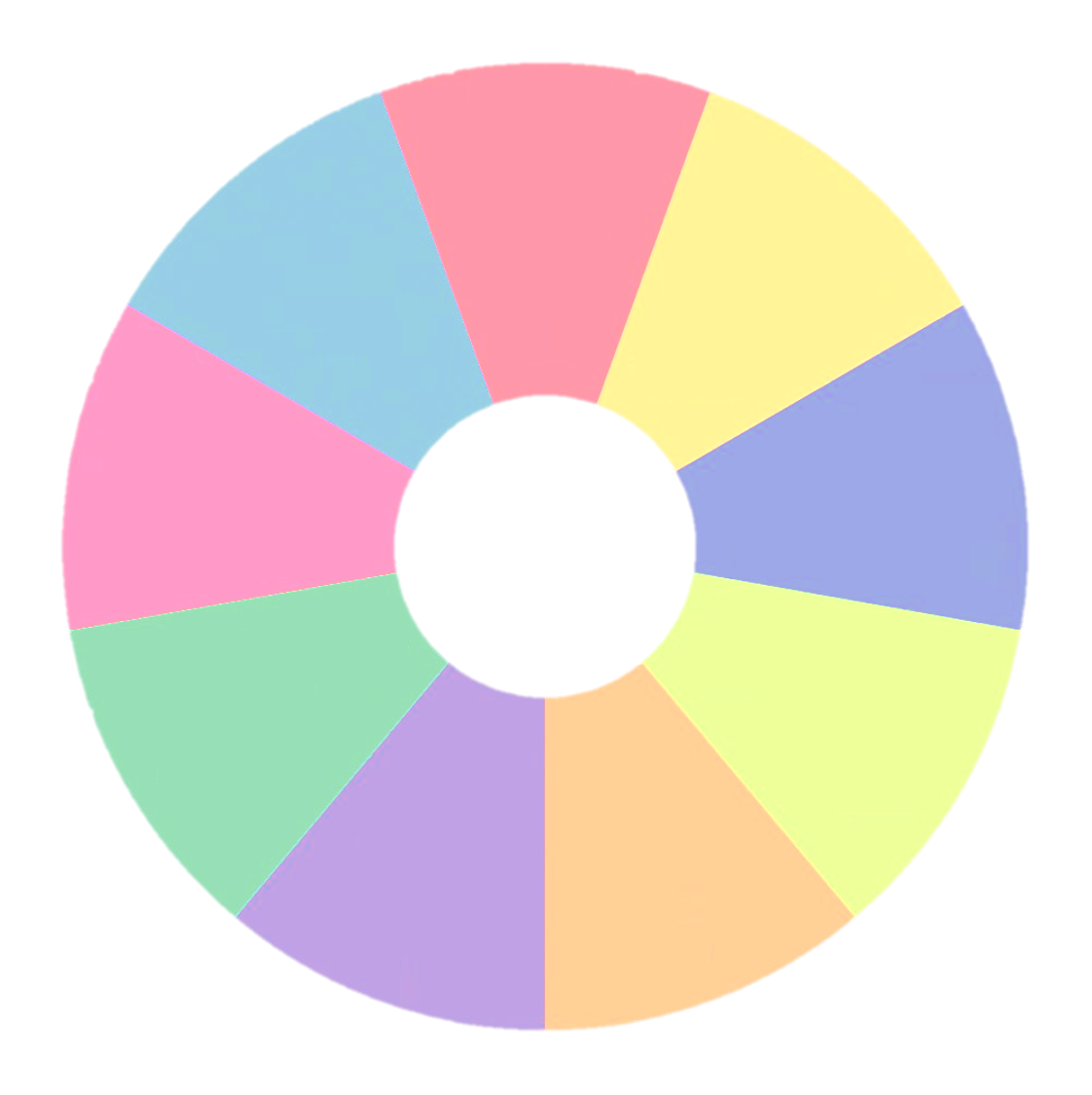


**Patient**

**& Caregiver**

**Antimicrobial**

**Stewardship**

**Pharmacist**

**Prescribers of**

**antimicrobials**

**Pharmacist**

**Administrator**

**Infection Control Practitioner**

**Physician Specialist**

**Antimicrobial Stewardship/ Infectious Diseases MD**

**Microbiologist**

**Nurse**

**Reference:**

1. Guideline consultation | Antimicrobial stewardship: systems and processes for effective antimicrobial medicine use | Guidance | NICE [Internet]. www.nice.org.uk. [cited 2023 Jan 3]. Available from: https://www.nice.org.uk/guidance/ng15/documents/antimicrobial-stewardship-guideline-consultation.

2. Antimicrobial stewardship programmes in health-care facilities in low - and middle -income countries [Internet]. www.who.int. Available from: https://www.who.int/publications/i/item/9789241515481.

3. Antimicrobial Stewardship - From Principles to Practice e-book [Internet]. The British Society for Antimicrobial Chemotherapy. [cited 2022 Dec 29]. Available from: https://bsac.org.uk/antimicrobial-stewardship-from-principles-to-practice-e-book/.

4. Chung GW, Wu JE, Yeo CL, et al. Antimicrobial stewardship: a review of prospective audit and feedback systems and an objective evaluation of outcomes. *Virulence*. 2013; **4:**151-157.

5. Jenkins JA, Pontefract SK, Cresswell K, Williams R, Sheikh A, Coleman JJ. Antimicrobial stewardship using electronic prescribing systems in hospital settings: a scoping review of interventions and outcome measures. JAC-Antimicrobial Resistance. 2022 May 3;4(3). ‌

6. Antimicrobial Stewardship | Public Health Ontario [Internet]. Public Health Ontario. 2018. Available from: https://www.publichealthontario.ca/en/health-topics/antimicrobial-stewardship ‌
